# Unveiling therapeutic targets and preventive components for kidney insufficiency and blood stasis-type BPH: bridging metabolomics, network pharmacology and reverse screening

**DOI:** 10.3389/fphar.2025.1584766

**Published:** 2025-06-19

**Authors:** Xiangpeng Kong, Haiqin Ren, Ke Pei, Yajun Yao, Zhigang Liang, Yunfeng Xue, Shuang Liang, Tongtong Li, Jing Zhang, Yuxia Guo, Miaorong Pei, Huifeng Li

**Affiliations:** ^1^ Institute of Pharmaceutical & Food Engineering, Chinese Medicine Master Studio of Wang Shimin, Shanxi University of Chinese Medicine, Jinzhong, China; ^2^ Shanxi Province Doctor Innovation Station (Shanxi Zhendong WuheTang Pharmaceutical Co., Ltd. Station), Changzhi, China; ^3^ Department of Chinese Medicine, Shanxi Pharmaceutical Vocational College, Taiyuan, China; ^4^ Department of Andrology, The Affiliated Hospital of Shanxi University of Chinese Medicine, Taiyuan, China; ^5^ The Third Clinical College, Shanxi University of Chinese Medicine, Jinzhong, China

**Keywords:** benign prostatic hyperplasia (BPH), kidney insufficiency and blood stasis, metabolomics, network pharmacology, integration, target, components, reverse screening

## Abstract

Benign prostatic hyperplasia (BPH) is a common disease affecting male urinary system function and quality of life, with its incidence increasing due to population ageing and unhealthy lifestyles. Modern medicine mainly adopts symptomatic treatments such as 5-alpha reductase inhibitors and alpha1 adrenergic receptor blockers. However, due to the complex pathogenesis of BPH, these drugs can only partially alleviate symptoms and have shortcomings such as high treatment costs and significant side effects. BPH is similar to the descriptions of “Jīng Lóng” and “Lóng Bì” in traditional Chinese medicine (TCM), and its onset is closely related to liver and kidney dysfunction. Kidney insufficiency and blood stasis are common clinical syndromes of BPH. Compared with modern medicine, treatment based on syndrome differentiation of TCM can achieve good results in treating this subtype of BPH. Therefore, guided by the holistic view of TCM, adopting a holistic and systematic research approach to explore therapeutic targets and potential therapeutic components for BPH with a specific syndrome can provide new ideas for the clinical treatment of BPH. This study integrated clinical metabolomics and network pharmacology to identify therapeutic targets for kidney insufficiency and blood stasis-type BPH. Serum analysis of BPH patients and healthy controls for testosterone, estradiol, SRD5α2, NF-κB p65, and TGF-β levels, alongside metabolomics and network pharmacology, revealed hormonal imbalance, increased inflammatory/fibrotic markers, and 58 differential metabolites in BPH. Pathway enrichment analysis identified 6 key metabolic pathways, while network pharmacology constructed four compound-reaction-enzyme-gene networks and pinpointed 178 potential targets, including 23 core targets. Reverse screening against the Yaozh Database-Natural Product AI Engine Platform matched 11 druggable targets with 49 interacting components, and target-component fitting analysis confirmed the reliability of 8 core targets. This combined approach validated the findings of hormonal imbalance and significant metabolic pathway changes and provided valuable insights for BPH treatment and drug development.

## Graphical Abstract

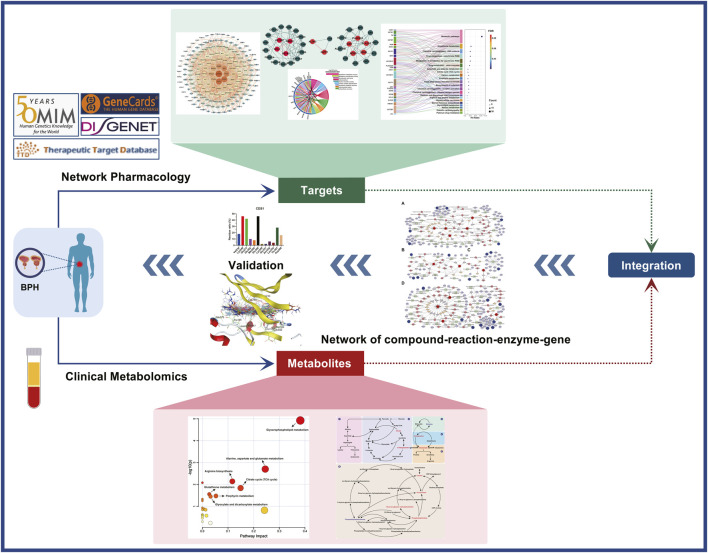

## 1 Introduction

Benign Prostatic Hyperplasia (BPH) is a common male disease characterised by abnormal hyperplasia of prostate tissue and often accompanied by symptoms such as urgency, frequent urination, incontinence, increased urination frequency, dysuria, and urinary retention (Infante Hernández et al., 2024). Its pathogenesis and mechanism are complex, and the risk factors involve metabolic diseases, hormonal imbalance disorders and chronic inflammation (Devlin et al., 2021; Untergasser et al., 2005; La Vignera et al., 2016). As a significant public health problem in the global ageing process, the prevalence of BPH increases significantly with age, and the incidence of BPH increases significantly after the age of 40 years (Lim, 2017). With the aggravation of global population ageing at present, along with people’s bad living habits and high-stress working environment, the incidence of BPH is showing an increasingly se upward trend (GBD 2019 Benign Prostatic Hyperplasia Collaborators, 2022). The lower urinary tract symptoms caused by the disease seriously impair the quality of life of patients, causing sleep disorders, anxiety, depression, and sexual dysfunction, and causing complications such as acute urinary retention and chronic kidney injury, resulting in the decline of the quality of life and work ability of patients. Long-term drug treatment and nursing costs pose continuous pressure on the social medical and health system and the families of patients (Carnevale et al., 2020). Modern treatment is based on α-receptor blockers and 5α-reductase inhibitors, but most of them have limitations such as insufficient drug response rates and long-term side effects (Wu et al., 2021; Strand et al., 2017; Halawani et al., 2024).

According to traditional Chinese medicine (TCM) theory, the pathogenesis of BPH is closely related to kidney insufficiency and blood stasis (Xu X. et al., 2022; Ma et al., 2023). With ageing, men gradually suffer from liver and kidney insufficiency and lack of qi and blood sources, resulting in qi stagnation, blood stasis, and phlegm dampness. Over time, it will congeal into hernia dysentery, accumulation in the lower abdomen and pudendal region, and develop urinary system diseases such as an adhesed bladder or uncontrolled bladder (Yong et al., 2015). In the clinical treatment of such diseases, TCM emphasises the coordination and compatibility of prescriptions, that is, the overall and comprehensive regulatory effect is achieved through the interaction of multiple components and targets (Zhao et al., 2022; Zhang et al., 2022). Modern precision medicine methods are also used to study traditional drugs and related mechanisms, and some achievements have been made (Li S. et al., 2024; Miao et al., 2023; Kong et al., 2023). Due to the differences in thinking between modern and traditional medical models, there are still many deficiencies in traditional medicine research based on modern precise models. For example, the reductionist approach focusing on a single target cannot capture the TCM compound’s systemic effect. At the same time, the efficacy screening based on modern isolation, *in vitro* and *in vivo* tests is time-consuming and expensive, and lacks the predictive power for multi-target synergy, which is difficult to translate into *in vivo* efficacy (Huo et al., 2022). This makes most mechanism research superficial, and it is difficult to analyse the interaction between endocrine, immune and metabolic pathways. Limitations have hindered the discovery of lead compounds of TCM with clinical translational value.

Reductionism and macro thinking are the most fundamental differences between modern precision medicine and traditional medicine models. The research model of single thinking is complex in revealing the pathogenesis of diseases, and new research ideas and methods are urgently needed (Welsby, 1999). Treating diseases is the common purpose of modern precision medicine and traditional medicine, both of which take tangible objective substances as treatment carriers. Therefore, under the guidance of the common purpose and treatment carrier of the two, research methods that can link the two thinking modes of precision and macro are introduced, which can not only avoid the one-sided thinking of reductionism, but also reflect the characteristics of macroscopic regulation of diseases to improve the effectiveness of drugs (Naaldenberg and Aarts, 2020). Modern systems pharmacology, omics and integration technology can provide a reference for the above research ideas (Li X. et al., 2024; Zhang et al., 2020). Network pharmacology can analyse the biological process of diseases and the coordinated action mechanism of multi-targets of drugs step by step by constructing a multi-dimensional interaction network of “disease-target/drug” (Li et al., 2021; Zhong et al., 2024). Based on the changes of endogenous metabolites in an organism, metabolomics accurately captures the characteristics of metabolic phenotypes and their dynamic responses to diseases, providing a molecular characterisation basis for traditional medicine’s “holistic view” (Sun et al., 2023; Yu et al., 2021; Jiang et al., 2023). The integrated analysis of network pharmacology-metabolome/transcriptome/proteome can reveal the spatiotemporal association between “microscopic molecular events” and “macroscopic pathological phenotypes” in the occurrence and development of diseases, and provide a quantifiable integrated analysis framework for targeted intervention of precision medicine and dialectical treatment of traditional medicine (Li X. et al., 2024; Zhang et al., 2020).

In recent years, with the rapid development of metabolomics and network pharmacology, reverse drug screening methods combined with artificial intelligence (AI) technology provide new concepts and tools for disease diagnosis and drug discovery for prevention and treatment (Ocana et al., 2025; Ata et al., 2024). Network pharmacology based on artificial intelligence can reveal the treatment mechanism of complex diseases from a large number of omics data, significantly improve the research efficiency of network pharmacology in traditional Chinese medicine in terms of network relationship mining, network target localization and network target navigation, and help the mining of biological basis and clinical value of TCM syndromes (Zhang et al., 2023). Machine learning and deep learning algorithms based on artificial intelligence can efficiently process and analyze large-scale biological data, integrate them into the whole drug development process, and accurately identify disease targets through multi-omics and network pharmacology analysis (Ai et al., 2021; Wang et al., 2023). For example, deep learning-based protein structure prediction (such as AlphaFold) can parse the 3D conformation of the target, supporting drug resistance assessment and structure-oriented design (Wu et al., 2024). AI-driven generative virtual screening has accelerated the development of novel molecular entities, combined with pharmacophores modelling to optimize pharmacokinetic/toxicological properties, and significantly shortened the preclinical development cycle (Gupta et al., 2021). The above application cases verify the translational potential of “multi-omics data, network pharmacology, and AI reverse design”. This integration strategy realises dimensionality reduction mining and network topology reconstruction of multi-dimensional biological big data through the deep intersection of systems biology and bioinformatics, which opens up a new path for elucidating the mechanism of complex disease heterogeneity and innovative drug development.

This study combined clinical metabolomics and network pharmacology with drug reverse screening to screen and verify potential therapeutic compounds for BPH with kidney insufficiency and blood stasis. Firstly, the metabolic fingerprint of BPH with kidney insufficiency and blood stasis was defined by clinical metabolomics, and then the BPH-specific target library was constructed by network pharmacology. On this basis, the differential endogenous metabolites obtained from the metabolomics study and the targets obtained from the network pharmacology study were imported into the Cytoscape software, respectively. The built-in plug-in Metscape was used to construct the metabolism-reaction-enzyme-gene network, and the core targets in the network were mined. The Yaozh Database-Natural Products AI Research and Development Platform compound reverse screening module was used to explore potential compounds against the above targets, and molecular fitting was performed to verify. The detailed technical roadmap is provided in Supplementary Figure S1.

## 2 Materials and methods

### 2.1 Materials

Acetonitrile (Batch number: 24100506G102) and methanol (Batch number: 21111019G112), both mass spectrometry grade, were purchased from Oceanpak Company. Formic acid (Batch number: H1913009), chromatography grade, was purchased from Shanghai Aladdin Bio-Chem Technology Co., LTD.

### 2.2 Samples Collection

Samples from patients with kidney insufficiency and blood stasis type BPH at Shanxi University of Chinese Medicine were collected and designated as the BPH group, while samples from healthy male individuals were collected and designated as the Control group. The baseline age data between the two groups did not exhibit a statistically significant difference (*P* > 0.05), indicating their comparability. The study was approved by the Institutional Ethics Committee (2022LL217). Blood samples of 5 mL were collected from each individual in the healthy and BPH groups, incubated at room temperature for 30 min, centrifuged at 3,000 rpm for 15 min at 4°C, and the serum was separated, aliquoted, and stored at −80°C for future use.

### 2.3 Serum biochemical indicators determination

The serum samples from each group were collected as specified in section 2.2. The levels of hormones such as testosterone (T) and estradiol (E2), as well as the tissue enzyme levels of steroid 5α-reductase 2 (SRD5α2), the affinity peptide of nuclear transcription factor-κB subunit p65 (NF-κB p65), and the tissue growth factors such as transforming growth factor β (TGF-β), were measured by using human-specific double-antibody sandwich enzyme-linked immunosorbent assay (ELISA) kits (T, Lot: 24110128H; E2, Lot: 24110120H; SRD5α2, Lot: 24110170H; NF-κB p65, Lot: 24110131H; TGF-β, Lot: 24110108H; Shanghai Kexing Trading Co. Ltd., China) according to the instructions provided with the Elisa kit. The absorbance values of the above indicators in each sample were measured by Rayto microplate reader (RT-6100, Shenzhen Leidu Life Science Co., Ltd.) at a wavelength of 450 nm. The standard curve regression equation was used for concentration conversion, and the accurate quantification of the target substance concentration in each sample was finally obtained.

### 2.4 Metabolomics study

#### 2.4.1 Metabolomics Sample preparation

100 μL of serum was taken from each sample in the Control and BPH groups, to which 300 μL of pre-cooled methanol (MS grade) was added to precipitate proteins. The mixture was then vortexed for 1 min and centrifuged at 13,000 rpm at 4°C for 15 min. The supernatant was collected and concentrated to dryness under a stream of nitrogen. The residue was reconstituted with 200 μL of methanol, vortexed for 3 min, and centrifuged again using the same method. The supernatant was collected to obtain the final sample. For each of the aforementioned processed samples, 3 μL was taken and mixed thoroughly, and this mixture was used as the Quality Control (QC) sample.

#### 2.4.2 Metabolic profile detection

5 μL of each sample solution from section 2.4.1 was injected into Ultra High Performance Liquid Chromatography (UHPLC)-MS/MS (Thermo Fisher Instruments Co.) for metabolic profiling analysis. Prior to the formal analysis of samples, QC samples were injected consecutively six times to equilibrate the system, and one QC sample was injected between every six samples to monitor the stability of the analytical system. Endogenous metabolites in serum were separated on an ACQUITY UPLC BEH C_18_ column (2.1 × 100 mm, 1.7 μm, Waters). The flow rate was controlled at 0.3 mL/min, with an injection volume of 5 μL and a column temperature of 40°C. The mobile phase was composed of acetonitrile (A) and 0.1% formic acid in water (B). The gradient elution program was as follows: 0.0–0.5 min, 5% A; 0.5–1.5 min, 5%–15% A; 1.5–4.5 min, 15%–30% A; 4.5–6.0 min, 30%–60% A; 6.0–9.0 min, 60%–70% A; 9.0–12.0 min, 70%–100% A; 12.0–13.0 min, 100% A; 13.0–13.5 min, 100%–5% A; 13.5–16.0 min, 5% A.

Electrospray ionization (ESI) mass spectrometry was acquired in both positive and negative ion modes. In positive ion mode, the spray voltage was set at 3.2 kV, with a sheath gas flow rate of 40 arb, an auxiliary gas flow rate of 5 arb, and an auxiliary gas heater temperature of 350°C. In negative ion mode, the spray voltage was adjusted to 2.5 kV, with a sheath gas flow rate of 38 arb, an auxiliary gas flow rate of 10 arb, and an auxiliary gas heater temperature of 300°C. The ion transfer tube temperature was maintained at 320°C, and the lens voltage (S-Lens RF Level) was set at 50 V. Full scan/data-dependent MS^2^ (Full MS/dd-MS^2^) was performed with a scan range of m/z 100 to 1,000. The first-stage mass resolution was 70,000 full width at half maximum (FWHM), and the second-stage resolution was 17,500 FWHM. The collision energy was set at 30 eV.

#### 2.4.3 Data preprocessing

The raw LC-MS/MS data files collected were imported into the Thermo Compound Discoverer v3.3 software to perform chromatographic peak alignment, peak filtering, peak extraction, and automatic integration processing. This generated multi-dimensional peak tables for both positive and negative ion modes, containing information such as relative molecular mass, retention time (RT), mass-to-charge ratio (m/z), and peak area. These peak tables were then used for peak annotation by comparing the secondary mass spectrometry information of the chromatographic peaks against databases such as mzCloud and ChemSpider, which include sub-databases like the Human Metabolome Database, Serum Metabolome Database, KEGG, and Metabolome Database. The obtained peak area data were normalized using total peak area normalization and subsequently imported into SIMCA-P v14.1 software for multivariate statistical analysis, including principal component analysis (PCA) and orthogonal partial least squares discriminant analysis (OPLS-DA). Differential metabolites were screened based on the constraints of a variable importance plot (VIP) value greater than 1 and a *P*-value less than 0.05.

Differential metabolites were matched to their potential chemical formulae by comparing their mass spectrometry data against the HMDB database (Human Metabolome Database; http://www.hmdb.ca/), with comparisons made between the primary and secondary mass spectrometry information to annotate further the obtained differential metabolites (Schymanski et al., 2014). The online analysis platform MetaboAnalyst 6.0 (https://www.metaboanalyst.ca/) was utilized to conduct pathway enrichment analysis on the differential metabolites, and a preliminary exploration of the relevant metabolic pathways was carried out in conjunction with the KEGG database (Kyoto Encyclopedia of Genes and Genomes; https://www.genome.jp/kegg/).

### 2.5 Metabolomics-network pharmacology integrated analysis

#### 2.5.1 Target screening

The potential targets associated with BPH, using the keywords “Prostatic Hyperplasia”, “Benign Prostatic Hyperplasia”, “Chronic benign prostatic hyperplasia”, and “Prostate enlargement”, were mined from OMIM Database (https://omim.org/), GeneCards Database (https://www.genecards.org/), TTD Database (http://db.idrblab.net/ttd/), and DisGeNET Database (http://www.disgenet.org). Additionally, the targets of candidate drugs for the clinical treatment of BPH were supplemented from DRUGBANK Database (https://go.drugbank.com/). All BPH-related targets from these five databases were combined, duplicates were removed, and a BPH disease target Database was constructed. These targets were then imported into Cytoscape v3.9.1 software, and the Metscape plugin was used to explore further potential targets involved in the metabolite-reaction-enzyme-gene network system that regulates BPH.

#### 2.5.2 Integrated analysis

The BPH-characteristic metabolites obtained from section 2.4 and the targets related to BPH metabolic regulation identified in section 2.5.1 were imported into the Metscape plugin of Cytoscape v3.9.1 software, respectively, to explore the targets that could participate in the regulation of BPH clinical phenotypes. The compound (metabolite)-reaction-enzyme-gene networks related to metabolite enrichment pathways were also established.

#### 2.5.3 Target interaction analysis

The String Database (https://string-db.org/) was employed to conduct protein-protein interaction (PPI) network analysis of the targets in section 2.5.1, and the results obtained were imported into Cytoscape v3.9.1 software to construct the PPI network. The core subnetworks were screened by employing the MCODE plugin in Cytoscape 3.9.1.

#### 2.5.4 Target enrichment analysis

The relevant targets associated with metabolic regulation in BPH under item 2.5.2 were imported into the DAVID Database (https://davidbioinformatics.nih.gov/) for Gene Ontology (GO) functional annotation and Kyoto Encyclopedia of Genes and Genomes (KEGG) pathway enrichment analysis. The species was set as “*Homo sapiens*”, and a screening criterion of *P* < 0.05 was applied. The obtained results were further sorted in ascending order based on the P value. Subsequently, the entries in Biological Process (BP), Cellular Component (CC), and Molecular Function (MF) categories of GO functional annotation, as well as the pathways in KEGG pathway enrichment, were selected. These results were visualized using the online bioinformatics analysis and visualization cloud platform of Wei Sheng Xin (http://www.bioinformatics.com.cn/).

### 2.6 Target-based component reverse screening and fitting validation

#### 2.6.1 Target-based component reverse screening

The regulatory targets for BPH phenotypes, which were integrated and analyzed under item 2.6, were imported into the Yaozh Database-Natural Products AI Research and Development Platform (https://npaiengine.yaozh.com/). Based on the AI algorithms of the Yaozh Database-Natural Product AI Engine Platform, activity predictions and rankings of candidate compounds were conducted.

#### 2.6.2 Target-component fitting validation

Based on the BPH phenotypic regulatory targets screened in section 2.6, the corresponding 3D protein structures were downloaded from the PDB (https://www.rcsb.org/) and Uniprot (https://www.uniprot.org/) databases. Subsequently, the protein structures were refined using the MOE software, and charges were added using the Amber10: EHT force field to identify suitable active pockets. Based on the lead compounds identified in Section 2.6.1, a target compound database was constructed by importing the data into the database module of MOE software (Chemical Computing Group, Inc., Montreal, Canada). The 3D structures of all compounds within the database were subsequently subjected to energy minimization. On this basis, a triangular matching position was adopted utilizing the docking module of MOE software, and the docking attempts for each compound were set to 10. With the LondondG and GBVI/WSA dG scoring rules, the compound database for each target was rigidly docked with the amino acid residue pockets of the target. The affinity between the screened lead compounds and the corresponding targets regulating BPH clinical phenotypes was verified through the fitting score of lead compound (ligand)-BPH phenotypic regulatory target (receptor), the protein-ligand interaction fingerprint (PLIF), and the 3D interaction target residue analysis of ligand-receptor.

### 2.7 Statistical analysis

Statistical analysis was conducted on the serum biochemical index data of item 2.3 using DPS software version 21.05 (Tang and Zhang, 2013). The measurement data that conformed to a normal distribution were expressed as Mean ± Standard deviation. An independent-sample t-test was employed to compare the significant differences between the model and healthy groups. When the homogeneity of variances was satisfied, the Student’s t-test was performed; otherwise, a corrected t-test was adopted. Statistical analysis of normalized metabolomics peak area data was conducted using GraphPad Prism v10 software, with significant differences between groups analyzed through the Mann-Whitney test. The experimental results are presented as Median ± Interquartile range (Median ± IQR), with *n* = 18. The significance level between BPH and Control groups was set at *α* = 0.05, and different levels of significance were denoted as *P < 0.05, **P < 0.01, ***P < 0.001, and ****P < 0.0001, respectively.

## 3 Results

### 3.1 Serum biochemical indicators analysis

**FIGURE 1:**
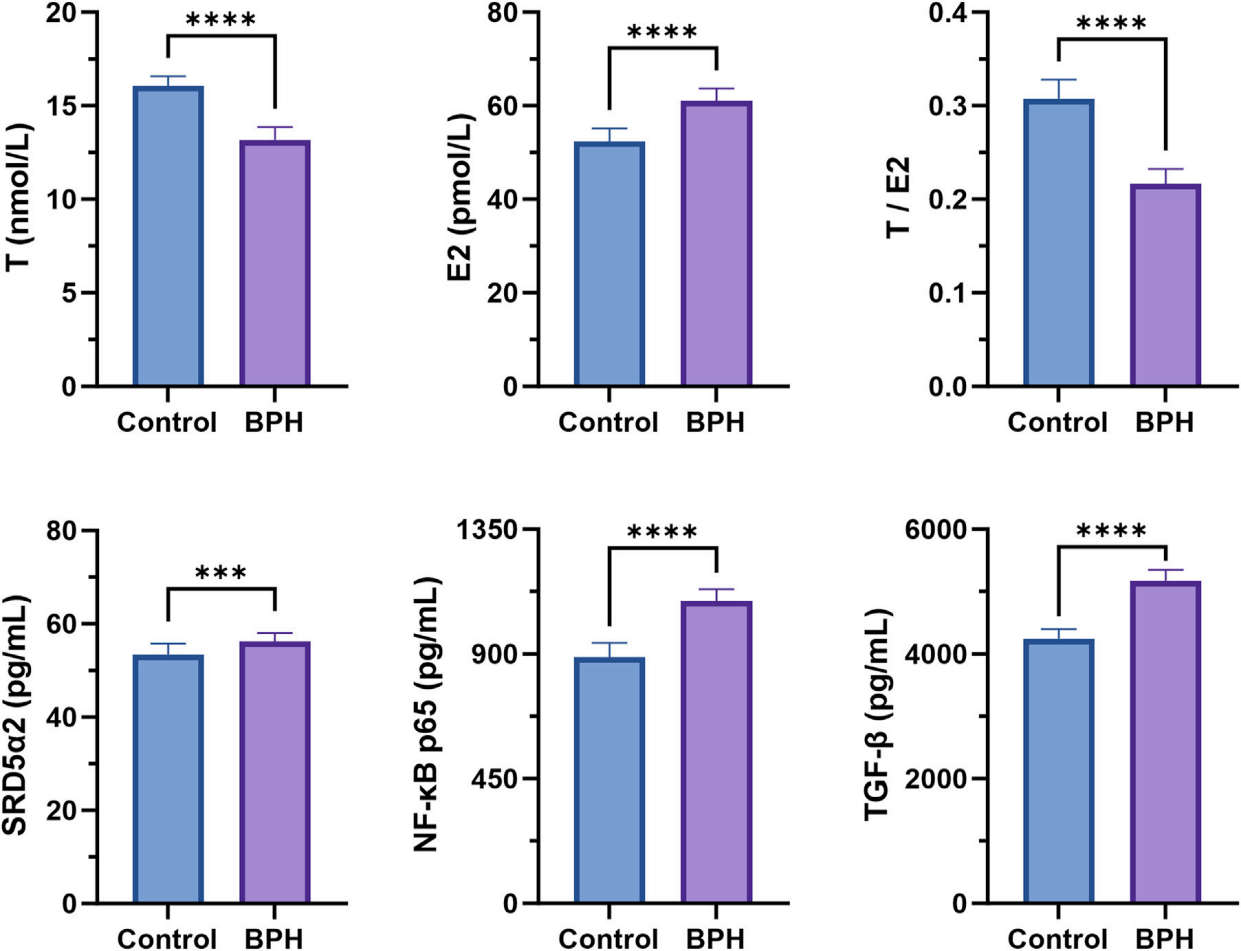
Serum biochemical indexes determination results. Control and BPH represent control and BPH groups, respectively. Data are presented as Mean ± standard deviation (*n* = 18 per group). Normality was assessed using the Shapiro-Wilk test (*P* > 0.05), and homogeneity of variances was evaluated by F test. Based on the assumptions, either Student’s t-test (equal variances) or Welch’s t-test (unequal variances) was applied to determine the significance of differences between groups. Effect sizes were calculated as Cohen’s d with 95% confidence intervals (CI). Significance levels were denoted as *P < 0.05, **P < 0.01, ***P < 0.001, and ****P < 0.0001.

The levels of various biochemical indicators in the serum of the healthy and BPH groups were determined according to the method described in section 2.2, as shown in Figure 1. The results indicated that, compared with the healthy group, the serum T level in the BPH group was significantly decreased (*P* < 0.001), while E2 was significantly increased (*P* < 0.001), and the T/E2 ratio was significantly decreased (*P* < 0.001). In addition, the tissue enzyme level of SRD5α2 exhibited an increasing trend (*P* < 0.001), and the nuclear factor NF-κB p65 and the transforming growth factor TGF-β also showed a significant upward trend (*P* < 0.001).

### 3.2 Metabolomics study

#### 3.2.1 Metabolic profile analysis

Metabolomic studies were conducted using the UHPLC-Q-Orbitrap HRMS technique. Taking QC samples as an example, the total ion chromatograms of serum samples in both positive and negative ion modes are presented in Supplementary Figure S2.

**FIGURE 2:**
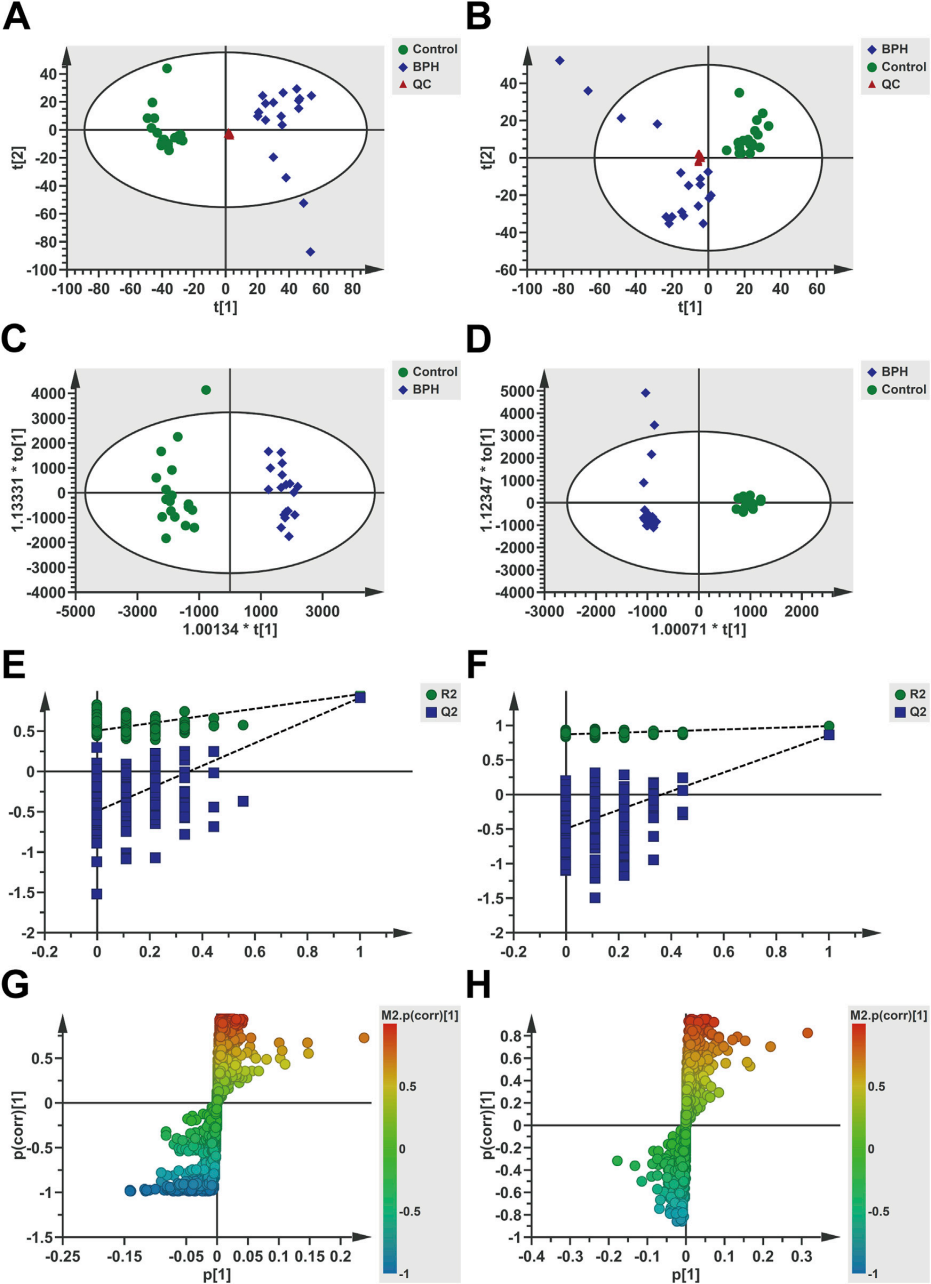
Multivariate statistical analysis of serum metabolomics under positive and negative ion modes. Positive ion mode **(A,C,E,G)** and Negative **(B,D,F,H)**. **(A,B)**: PCA score scatter plots, **(C,D)**: OPLS-DA score scatter plots, **(E,F)**: Permutation test results (200 iterations), **(G,H)**: S-plots. t [1] and t [2] represent the first and second principal component scores, respectively, with scatter points corresponding to different groups.

**FIGURE 3:**
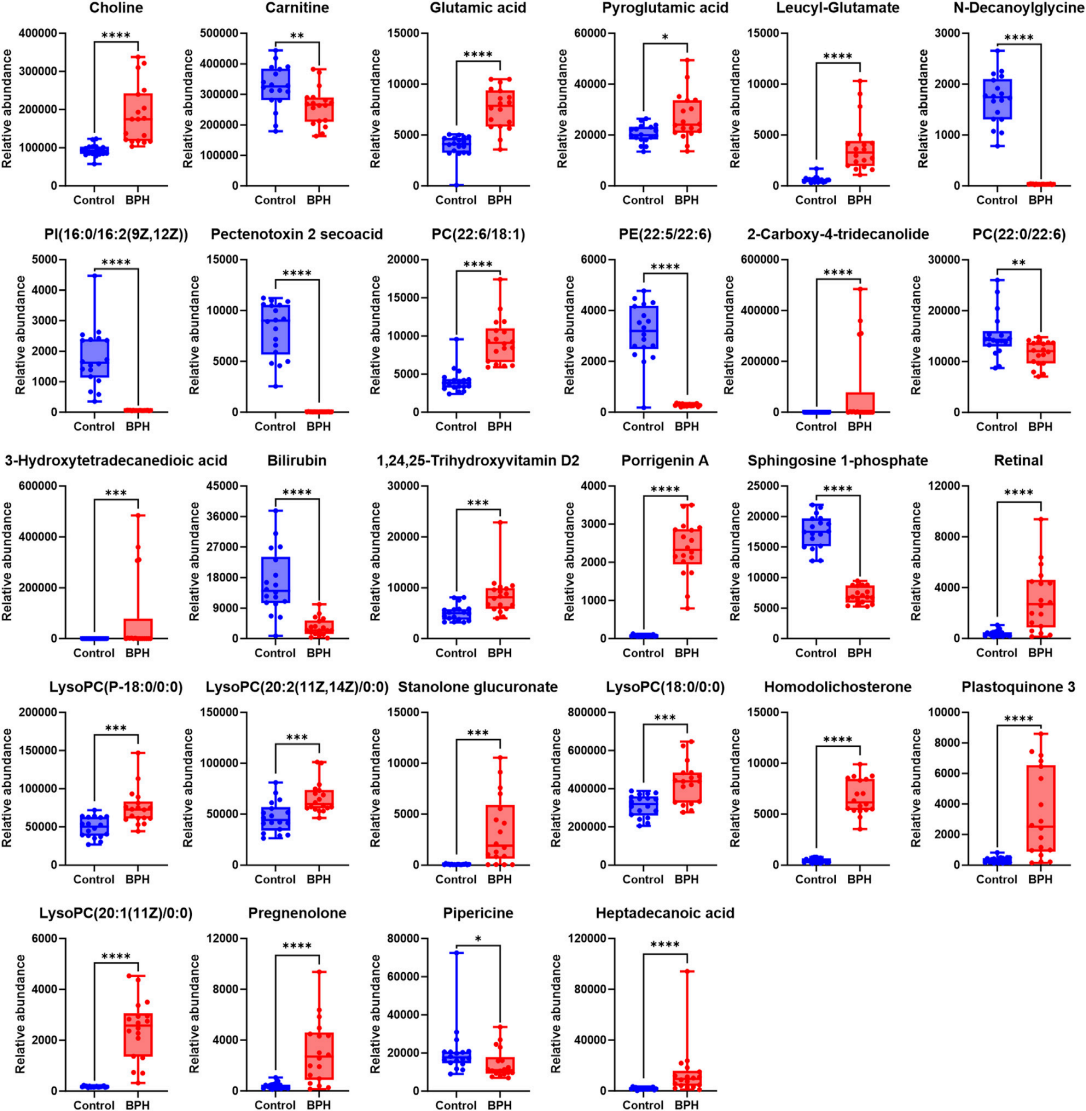
Normalized Peak Areas of Differential Metabolites under Positive Ion Mode. Control and BPH represent control and BPH groups, respectively. Data are presented as median ± interquartile range (IQR; *n* = 18 per group). The Mann-Whitney U test was used to assess the significance of differences between groups, as the data were non-normally distributed (Shapiro-Wilk test, *P* ≤ 0.05). Effect sizes were calculated as Cohen’s d with 95% confidence intervals (CI). Significance levels were denoted as *P < 0.05, **P < 0.01, ***P < 0.001, and ****P < 0.0001.

Multivariate statistical analysis was conducted on serum metabolomics data, with the results presented in Figure 2. As evident from the PCA analysis results (Figures 2A,B), the QC samples clustered well in both positive and negative ion modes, indicating the stability and reliability of the analytical method, and the identified differential metabolites were able to reflect the biological differences among samples. The samples from the Control and BPH groups were clearly distinguished, and the samples within each group clustered well within a specific range, suggesting significant differences in the serum endogenous metabolic profiles between BPH patients and healthy individuals. Further OPLS-DA analysis results showed (Figures 2C,D) that the model parameters were *R* ^*2*^ *Y* = 0.96 and *Q* ^*2*^ = 0.915 in positive ion mode and *R* ^*2*^ *Y* = 0.99 and *Q* ^*2*^ = 0.86 in negative ion mode. *R* ^*2*^ *Y* and *Q* ^*2*^ were close to 1 in both modes, indicating reasonable model interpretation and prediction. The Permutation test results in positive and negative ion modes (Figures 2E, 3F) showed that the *R* ^*2*^ and *Q* ^*2*^ points on the left were lower than the original *R* ^*2*^ and *Q* ^*2*^ values on the right, and the regression line of the *Q* ^*2*^ points intersected the vertical axis below the origin, indicating that the model was not overfitted and the model validation was passed. S-plot diagrams were drawn (Figures 2G,H), in which the scatter points in the upper left corner and the lower right corner represented metabolites with significant differences between the two groups.

#### 3.2.2 Differential metabolite analysis

Statistical analysis was conducted on the normalized peak area data for the two groups to evaluate significant differences. With reference to the S-plot scatter plot, differential metabolites were screened based on the VIP value (VIP >1) and significance test results (*P* < 0.05) as constraints. After annotation by comparison with the HMDB database, a total of 58 differential metabolites were obtained, mainly including organic acids and their esters, phospholipids, amino acids and dipeptides, steroids and their derivatives, prostaglandins, vitamins, and lactones, as shown in Supplementary Table S1.

**FIGURE 4:**
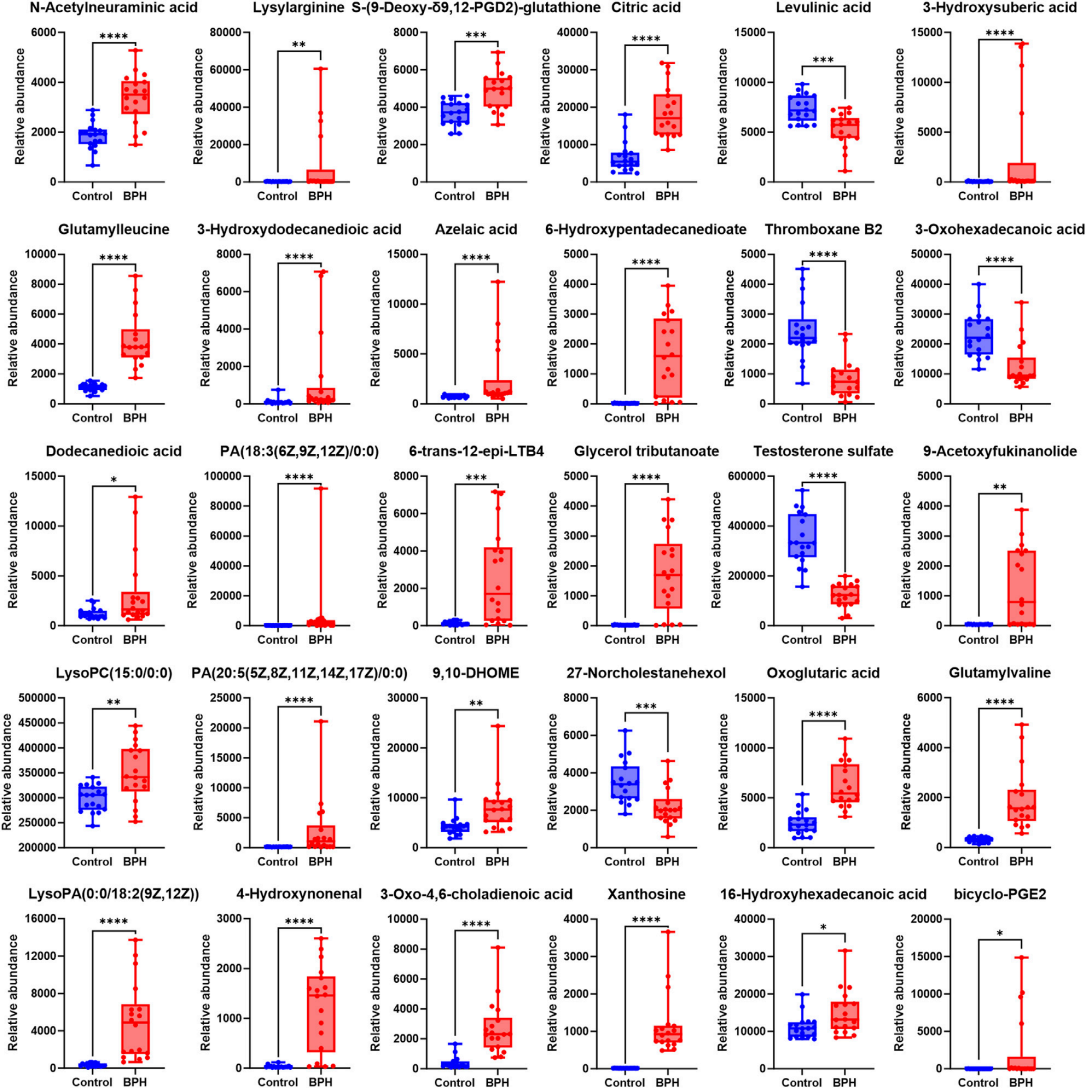
Normalized Peak Areas of Differential Metabolites under Negative Ion Mode. Control and BPH represent control and BPH groups, respectively. Data are presented as median ± interquartile range (IQR; *n* = 18 per group). The Mann-Whitney U test was used to assess the significance of differences between groups, as the data were non-normally distributed (Shapiro-Wilk test, *P* ≤ 0.05). Effect sizes were calculated as Cohen’s d with 95% confidence intervals (CI). Significance levels were denoted as *P < 0.05, **P < 0.01, ***P < 0.001, and ****P < 0.0001.

To further clarify the relative levels of the aforementioned metabolites in the BPH patients compared to the healthy population, the normalized peak areas of the selected metabolites were imported into GraphPad Prism v10 software to generate box plots, as shown in Figures 3, 4. The results indicated that, compared to the Control group, the relative levels of 44 metabolites, including glutamic acid and pyroglutamic acid, the dipeptides glutamylvaline, leucyl-glutamate, glutamylleucine, and lysylarginine, the organic acids and esters oxoglutaric acid, citric acid, 3-hydroxysuberic acid, 3-hydroxydodecanedioic acid, 3-hydroxytetradecanedioic acid, 16-hydroxyhexadecanoic acid, azelaic acid, dodecanedioic acid, 6-hydroxypentadecanedioate, 9,10-DHOME, the phospholipids PC (22:6/18:1), LysoPC (15:0/0:0), LysoPC (20:1 (11Z)/0:0), LysoPC (P-18:0/0:0), LysoPC (20:2 (11Z, 14Z)/0:0), LysoPC (18:0/0:0), PA (18:3 (6Z, 9Z, 12Z)/0:0), PA (20:5 (5Z, 8Z, 11Z, 14Z, 17Z)/0:0), LysoPA (0:0/18:2 (9Z, 12Z)), the vitamins retinal and 1,24,25-Trihydroxyvitamin D2, the steroids stanolone glucuronate, homodolichosterone, pregnenolone, the lactones 9-acetoxyfukinanolide and 2-carboxy-4-tridecanolide, and choline, N-acetylneuraminic acid, 4-hydroxynonenal, heptadecanoic acid, glycerol tributanoate, 6-trans-12-epi-LTB4, bicyclo-PGE2, S-(9-Deoxy-δ9,12-PGD2)-glutathione, xanthosine, 3-oxo-4,6-choladienoic acid, plastoquinone 3, porrigenin A, were significantly elevated in the serum of the BPH group. Conversely, the relative levels of 14 metabolites, including levulinic acid and 3-oxohexadecanoic acid, the phospholipids PI(16:0/16:2 (9Z, 12Z)), PE (22:5/22:6), PC(22:0/22:6), the steroids testosterone sulfate and 27-norcholestanehexol, and the compounds carnitine, N-decanoylglycine, pectenotoxin 2 secoacid, thromboxane B2, bilirubin, sphingosine 1-phosphate, pipericine, were significantly reduced.

#### 3.2.3 Metabolic pathway analysis

**FIGURE 5:**
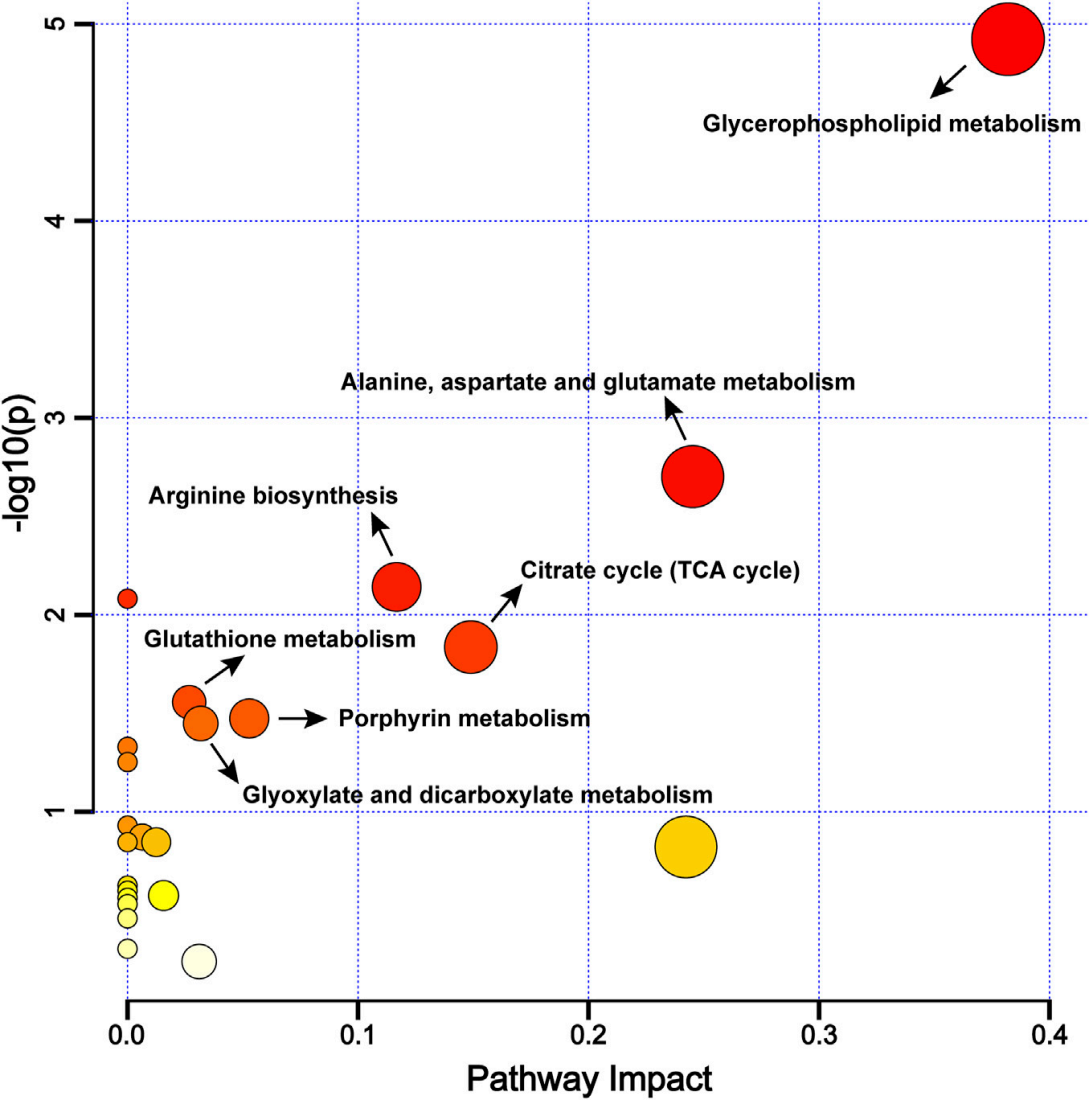
Metabolic Pathway Enrichment Bubble Chart. Note: The larger the -log10(P) value, the redder the bubble color, indicating a higher significance of the metabolic pathway; the more significant the Impact value, the bigger the bubble, indicating a more significant overall influence of the metabolic pathway.

**FIGURE 6:**
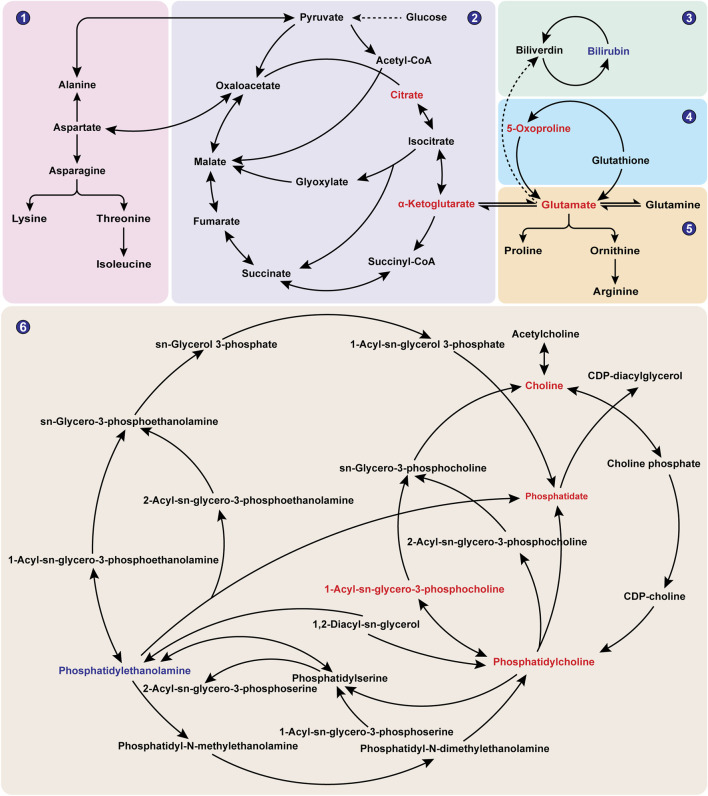
Network Diagram of BPH-Related Metabolites. Note: Red indicates metabolites with significantly elevated levels in the BPH group, while blue indicates metabolites with significantly reduced levels in the BPH group. ①Alanine, aspartate and glutamate metabolism; ②Citrate cycle (TCA cycle); ③Porphyrin metabolism; ④Glutathione metabolism; ⑤Arginine biosynthesis; ⑥Glycerophospholipid metabolism.

To further explore the differential metabolic pathways between BPH patients and healthy individuals, the HMDB numbers of the screened metabolites were imported into the MetaboAnalyst 6.0 online analysis platform, combined with the KEGG database for metabolic pathway enrichment and metabolite network construction. A bubble plot was drawn with the pathway impact factor (Impact) as the abscissa and the P-value as the ordinate, as shown in Figure 5. A metabolite network diagram was constructed based on the important pathways involved by differential metabolites, as shown in Figure 6. The results revealed a total of 23 enriched metabolic pathways. Potential target pathways were further screened based on the conditions of Impact ≥0.01 and *P* < 0.05, yielding glycerophospholipid metabolism, alanine, aspartate and glutamate metabolism, arginine biosynthesis, citrate cycle (TCA cycle), glutathione metabolism, porphyrin metabolism, and glyoxylate and dicarboxylate metabolism. It suggests that the occurrence of BPH may be related to these pathways. As shown in Figure 6, the screened biomarkers played important roles in pathways such as glycerophospholipid metabolism, the TCA cycle, glutathione metabolism, and porphyrin metabolism.

### 3.3 Metabolomics-network pharmacology integrated analysis

#### 3.3.1 Target screening

The keywords “Prostatic Hyperplasia”, “Benign Prostatic Hyperplasia”, “Chronic benign prostatic hyperplasia” and “Prostate enlargement” were searched in the OMIM database (Online Mendelian Inheritance in Man; https://omim.org/), resulting in the identification of 1,145 targets. Based on the median rule (Sun et al., 2024), quickq extracted 1,606 targets (with Relevance scores exceeding 10.5117 for Prostatic Hyperplasia, 14.6494 for Benign Prostatic Hyperplasia, 22.7554 for Chronic benign prostatic hyperplasia, and 0.2315 for Prostate enlargement). Additionally, 12 targets were obtained from TTD, and 331 (with scores exceeding 0.02) were retrieved from DisGeNET. Furthermore, 96 drug targets for clinical conditions, including prostatic hyperplasia, benign prostatic hyperplasia, chronic prostatic hyperplasia, and prostate enlargement, were supplemented from DRUGBANK. A BPH disease target database containing 1,197 targets was established by merging these five databases and removing duplicates. These 1,197 targets were then imported into the Metscape plugin of Cytoscape v3.9.1 for further analysis, yielding 178 BPH-related targets capable of participating in the metabolite-reaction-enzyme-gene network regulation.

#### 3.3.2 Integrated analysis

**FIGURE 7:**
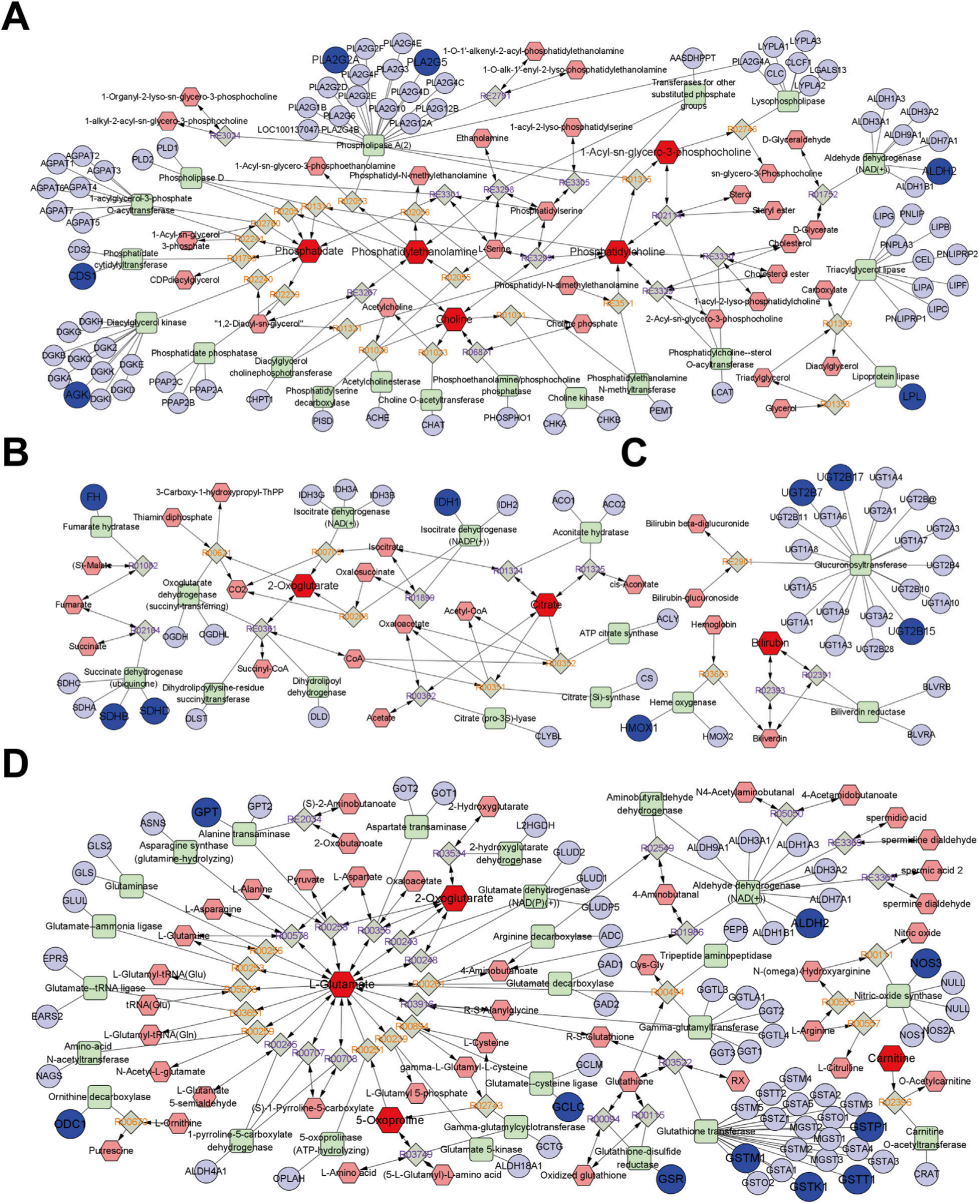
The network of compound-reaction-enzyme-gene. **(A)** Glycerophospholipid metabolism pathway; **(B)** TCA cycle; **(C)** Porphyrin metabolism; **(D)** Uric acid cycle and amino acid metabolism. Red hexagons indicate compounds, grey diamonds indicate reactions, green squares indicate enzymes, and blue circles indicate genes. The darker shapes represent the BPH characteristic metabolites (dark red) and BPH-related targets (dark blue).

Through further integrated analysis, Metscape obtained compound-reaction-enzyme-gene networks for glycerophospholipid metabolism, the TCA cycle, porphyrin metabolism, uric acid cycling, and amino acid metabolism, respectively, as shown in Figure 7. Among them, targets such as AGK, CDS1, ALDH2, PLA2G2A, PLA2G5, and LPL were found to participate in glycerophospholipid metabolism through endogenous metabolites including Phosphatidate, Phosphatidylethanolamine, Choline, Phosphatidylcholine, and 1-Acyl-sn-glycero-3-phosphocholine (Figure 7A). Targets such as SDHB, SDHD, IDH1, and FH were able to regulate the TCA cycle through metabolites like citrate and 2-Oxoglutarate (Figure 7B). Targets such as UGT2B15, UGT2B7, UGT2B17, and HMOX-1 could directly or indirectly regulate porphyrin metabolism through endogenous metabolites, including Bilirubin (Figure 7C). Additionally, targets such as GPT, ODC1, ALDH2, NOS3, GCLC, GSR, GSTT1, GSTK1, GSTM1, and GSTP1 were found to regulate uric acid cycling and amino acid metabolism through endogenous metabolites like 2-Oxoglutarate, L-Glutamate, 5-Oxoproline, and Carnitine (Figure 7D).

#### 3.3.3 Target interaction analysis

**FIGURE 8:**
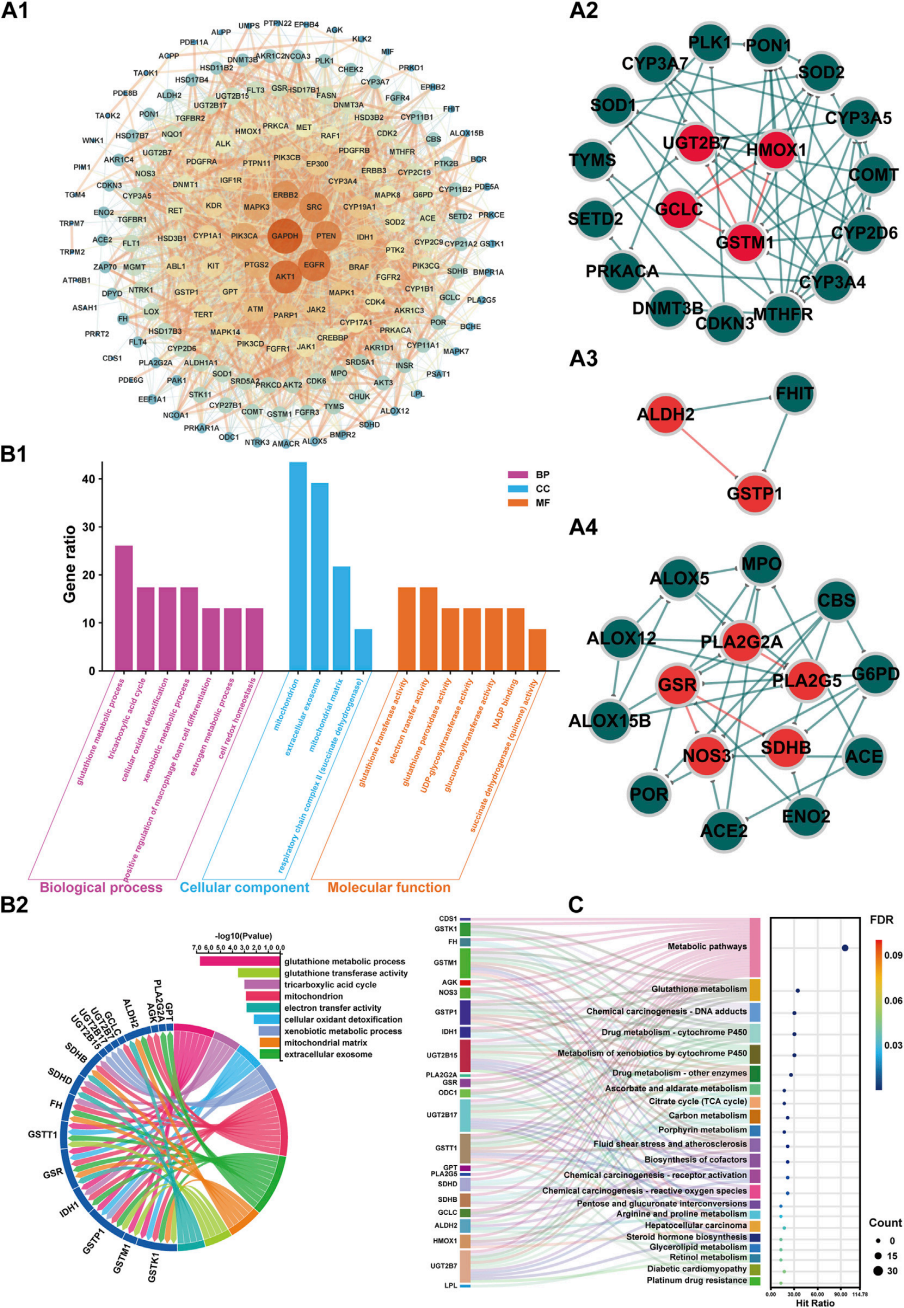
PPI network, GO, and KEGG enrichment analysis of BPH-associated targets in the metabolite-enzyme-gene network. **A1**: PPI network of 178 BPH-associated targets in the metabolite-enzyme-gene network. **A2**: Core subnetwork (Cluster 1, Score: 5.778). **A3**: Core subnetwork (Cluster 2, Score: 4.571). **A4**: Core subnetwork (Cluster 3, Score: 3.000). In the PPI network **(A1)**, the nodes represent protein targets associated with BPH, with the color and size of the nodes adjusted according to their degree values, where a higher degree value indicates more interactions between the protein and other proteins in the network. The edges represent the interaction relationships between proteins, with the thickness of the edges indicating the strength of the interactions, where thicker edges represent stronger interactions. In the core subnetworks **(A2–4)**, red nodes denote integrated targets while green nodes represent non-integrated targets. Similarly, red edges indicate interaction relationships between integrated targets, whereas green edges show interactions among non-integrated targets. **B1**: Plot illustrating the biological processes, cellular components, and molecular functions associated with the integrated targets from Gene Ontology (GO) term enrichment analyses (*P* < 0.05). **B2**: Correlation chord diagram of GO terms (Gene ratio >15) with integrated targets. **C**: The KEGG pathways Sankey diagram of integrated targets.

On this basis, the 178 screened BPH targets were imported into the String database, with the species set to “*Homo sapiens*”. The default confidence level of 0.40 was selected for the Minimum required interaction score (Szklarczyk et al., 2021; Menche et al., 2015; Cheng et al., 2018), and isolated nodes were removed to obtain these targets’ PPI information. Topological analysis was conducted using Cytoscape v3.9.1 software to construct the PPI network, as shown in Figure 8A1. Building on this foundation and incorporating the integrated target findings from section 3.3.2, the MCODE plugin in Cytoscape 3.9.1 was utilized to mine PPI core subnetworks, ultimately identifying seven functional subnetworks with significant connectivity. Among the subnetwork clusters, 3 core subnetworks had high overlap with the integration targets of item 3.3.2, see Figure 8A2 (overlaped with integrated targets UGT2B7, HMOX1, GCLC and GSTM1), Figure 8A3 (overlaped with integrated targets ALDH2 and GSTP1) and Figure 8A4 (overlaped with integrated targets PLA2G2A, PLA2G5, GSR, SDHB and NOS3).

#### 3.3.4 Target enrichment analysis

GO and KEGG enrichment analyses were conducted on integrated BPH-related target genes from section 3.3.2. The BP, CC, and MF terms ranked by GO annotation with *P* < 0.05 were annotated, as shown in Figure 8B1. The results indicated that BP primarily encompassed biological processes such as glutathione metabolic process, tricarboxylic acid cycle, cellular oxidant detoxification, xenobiotic metabolic process, positive regulation of macrophage foam cell differentiation, estrogen metabolic process, and cell redox homeostasis. CC mainly included cellular components such as mitochondrion, extracellular exosome, mitochondrial matrix, and respiratory chain complex II (succinate dehydrogenase). MF primarily encompassed glutathione transferase activity, electron transfer activity, glutathione peroxidase activity, UDP-glycosyltransferase activity, glucuronosyltransferase activity, NADP binding, succinate dehydrogenase (quinone) activity.

Furthermore, the correlation analysis presented in Figure 8B2 between the key items of GO enrichment analysis and the integration targets under item 3.1.2 reveals a network of interconnected pathways involving glutathione metabolism (GSTK1/GSTM1/GSTP1/GSR), mitochondrial energetics (TCA cycle: IDH1/SDHB/SDHD/FH; respiratory complex II activity), xenobiotic/estrogen detoxification (UGT2B/GSTT1), cellular redox homeostasis (GSR/ALDH2), and extracellular exosome components (GSTK1/IDH1). Key multifunctional nodes (GSTK1, IDH1, SDHB/SDHD) integrate mitochondrial matrix functions, oxidant detoxification, and electron transfer, while UGT2B isoforms and GSTP1 highlight phase II metabolism. Enriched terms like macrophage foam cell differentiation and NADP binding suggest broader implications in metabolic-inflammatory crosstalk and redox cofactor utilization. This multi-pathway synergy underscores therapeutic potential in oxidative stress-related pathologies, neurodegenerative disorders, and drug metabolism variability.

The KEGG enrichment analysis presented in Figure 8C underscores shared functional hubs across pathways, with GSTK1/GSTM1/GSTP1/GSTT1, UGT2B15/UGT2B17/UGT2B7, IDH1, and SDHB/SDHD acting as multipath regulators. These hubs converge on glutathione-dependent detoxification (Glutathione/Drug/Chemical carcinogenesis pathways), mitochondrial bioenergetics (Citrate cycle/Carbon metabolism), and xenobiotic-hormone crosstalk (Steroid/Retinol metabolism), bridging oxidative stress mitigation (GSR/HMOX1), metabolic-epigenetic dysregulation (IDH1), and carcinogen/drug processing (GST/UGT2B isoforms). Notably, GSTK1 and UGT2B subfamily members co-regulate Chemical carcinogenesis-DNA adducts, Drug metabolism-Cytochrome P450, and extracellular exosome signaling, while IDH1/SDHB/SDHD link TCA cycle defects to redox imbalance in diabetic cardiomyopathy and tumorigenesis. These multifunctional nodes highlight conserved targets for therapeutic intervention in oxidative stress-driven pathologies, chemoresistance, and metabolic-inflammatory diseases.

### 3.4 Target-based component reverse screening and fitting validation

#### 3.4.1 Target-based component reverse screening

The 23 phenotypic regulatory targets involved in the clinical metabolome of BPH from section 3.3.2 were imported into the Yaozh Database-Natural Product AI Engine Platform. This resulted in the identification of 11 human druggable targets, namely, ALDH2, CDS1, ODC1, IDH1, NOS3, PLA2G2A, SDHB, UGT2B7, HMOX1, PLA2G5, and GSR. Based on the platform’s AI algorithms, screening criteria were established: ligands targeting the targets were ranked in the top 10, with a minimum of one target hit per ligand, and druggability was filtered for Drug-like properties. Finally, 49 compounds with potential interactions with the aforementioned 11 targets were reverse-screened and presented in Table 1.

**TABLE 1:** List of compounds identified through reverse screening based on targets.

ID	Compounds name	Predicted level	Average molecular ranking	Bioavailability	Log P	Targets
1	2-[(4-methyl-2-oxo-2H-chromen-7-yl)oxy]acetonitrile	3-HIGH	1	51.2416	2.0038	ALDH2
2	Prunetin	3-HIGH	1	51.2911	2.8798	
3	methyl 2-[(4-methyl-2-oxo-2H-chromen-7-yl)oxy]propanoate	3-HIGH	1	51.7072	2.0417	
4	14,15-dimethyl-9,13-dioxatetracyclo [8.7.0.0^2^,^7^.0^12^,^16^]heptadeca-1(17),2(7),3,5,10,12 (16),14-heptaen-8-one	3-HIGH	1	45.7279	4.3092	
5	13,14-dimethyl-8,12-dioxatetracyclo [7.7.0.0^2^,^6^.0^11^,^15^]hexadeca-1(16),2(6),9,11(15),13-pentaen-7-one	3-HIGH	1	47.3155	3.6447	
6	3,4,8,9-tetramethyl-7H-furo [2,3-f]chromen-7-one	3-HIGH	1	43.317	3.7729	
7	2,3,5,6-tetramethyl-7H-furo [3,2-g]chromen-7-one	3-HIGH	1	50.3881	3.7729	
8	3,5-dimethyl-6-propyl-7H-furo [3,2-g]chromen-7-one	3-HIGH	1	39.1559	4.1085	
9	14,15-dimethyl-9,13-dioxatetracyclo [8.7.0.0^2^,^7^.0^12^,^16^]heptadeca-1(17),2(7),10,12(16),14-pentaen-8-one	3-HIGH	1	46.7	4.0348	
10	2,3-dimethyl-5-propyl-7H-furo [3,2-g]chromen-7-one	3-HIGH	1	42.3293	4.1085	
11	6-ethyl-2,3,5-trimethyl-7H-furo [3,2-g]chromen-7-one	2-MED	2	42.7024	4.0269	
12	2,3,5-trimethyl-6-(propan-2-yl)-7H-furo [3,2-g]chromen-7-one	2-MED	3	41.466	4.5879	
13	15,16-dimethyl-10,14-dioxatetracyclo [9.7.0.0^2^,^8^.0^13^,^17^]octadeca-1(18),2(8),11,13(17),15-pentaen-9-one	2-MED	6	45.5718	4.4249	
14	5-butyl-2,3-dimethyl-7H-furo [3,2-g]chromen-7-one	2-MED	9	39.4772	4.4986	
1	2-Bromo-4-(5-Hydroxy-2-Imino-3H-Imidazol-4-Ylidene)-1H,5H,6H,7H-Pyrrolo [2,3-C]Azepin-8-One	3-HIGH	2	66.1292	0.0663	CDS1
2	2-Bromo-4-[(4Z)-5-Hydroxy-2-Imino-3H-Imidazol-4-Ylidene]-1H,5H,6H,7H-Pyrrolo [2,3-C]Azepin-8-One	3-HIGH	3	64.5438	1.1163	
3	2-Imino-5-{8-Oxo-1H,5H,6H,7H-Pyrrolo [2,3-C]Azepin-4-Ylidene}Imidazolidin-4-One	2-MED	4	61.3411	−0.6962	
4	2-Bromo-4-[(4E)-5-Hydroxy-2-Imino-3H-Imidazol-4-Ylidene]-1H,5H,6H,7H-Pyrrolo [2,3-C]Azepin-8-One	2-MED	5	64.5438	1.1163	
5	5-{3-Bromo-8-Oxo-1H,5H,6H,7H-Pyrrolo [2,3-C]Azepin-4-Ylidene}-2-Iminoimidazolidin-4-One	2-MED	7	65.4907	0.0663	
6	2-Bromo-4-[(4Z)-2,5-Dihydroxyimidazol-4-Ylidene]-1H,5H,6H,7H-Pyrrolo [2,3-C]Azepin-8-One	2-MED	8	63.733	1.5059	
1	8,8-Dimethyl-3-(2,4,5-Trimethoxyphenyl)-2H,3H-Pyrano [2,3-F]Chromen-4-One	3-HIGH	1	42.9611	4.2555	ODC1
2	Pongachalcone Ii	3-HIGH	1	43.466	4.1867	
3	Isobavachromene	3-HIGH	1	48.7957	4.1781	
4	5-Methoxy-2,2-dimethyl-7-[2-(4-hydroxyphenyl)ethenyl]-2H-1-benzopyran	3-HIGH	1	46.0978	4.7554	
5	5-Methoxy-2,2-dimethyl-7-[2-(4-hydroxy-3-methoxyphenyl)ethenyl]-2H-1-benzopyran	3-HIGH	1	45.5547	4.764	
1	Gamma-Mangostin	3-HIGH	1	31.8231	4.786	IDHC
1	Ent-Epicatechin	3-HIGH	1	28.1641	1.5461	NOS3
1	Bolinaquinone	3-HIGH	1	40.2143	4.6695	PA2GA
1	(1R,2S,7S,8S,9S,10S)-2,6,6,9-Tetramethyltetracyclo [5.4.0.0^2^,^9^.0^8^,^10^]Undecane	3-HIGH	1	54.5271	4.1048	UD2B7
2	(1R,2R,7S,9S)-3,3,7-Trimethyl-8-Methylidenetricyclo [5.4.0.0^2^,^9^]Undecane	3-HIGH	1	46.5439	4.415	
3	(1aR,4S,4aR,7S,7aS,7bS)-1,1,4,7-tetramethyl-2,3,4a,5,6,7,7a,7b-octahydro-1aH-cyclopropa [e]azulen-4-ol	3-HIGH	2	50.552	3.4657	
4	(1R,2S,7S,8S,9R)-2,6,6,9-Tetramethyltricyclo [5.4.0.0^2^,^9^]Undecan-8-Ol	3-HIGH	3	47.9194	3.6098	
5	(1R,2S,7S,8R,9R)-2,6,6,9-Tetramethyltricyclo [5.4.0.0^2^,^9^]Undecan-8-Ol	3-HIGH	4	47.9194	3.6098	
6	(1S,2R,5S,6S,7S,8R)-1,5-Dimethyl-8-(Prop-1-En-2-Yl)Tricyclo [5.3.0.0^2^,^6^]Decane	3-HIGH	5	40.4694	4.2709	
7	(1S,2R,5S,6S,7S,8S)-1,5-Dimethyl-8-(Prop-1-En-2-Yl)Tricyclo [5.3.0.0^2^,^6^]Decane	2-MED	6	40.4694	4.2709	
8	(+)-Ledol	2-MED	7	50.552	3.4657	
9	(1S,2R,7S,8R,9S)-2,6,6,9-tetramethyltricyclo [5.4.0.02,8]undecan-9-ol	2-MED	8	50.3899	3.6098	
10	(1S,2S,7S,8S)-2,6,6,9-Tetramethyltricyclo [5.4.0.0^2^,^8^]Undec-9-Ene	2-MED	9	45.1124	4.415	
11	Epiglobulol	2-MED	10	50.552	3.4657	
1	1-benzyl-1H-imidazole	1-LOW	2	55.807	1.9314	HMOX1
2	Climbazole	1-LOW	2	61.0545	3.7293	
3	2-(1H-imidazol-1-yl)acetic acid	1-LOW	2	74.7304	−0.0323	
4	3-{4-[(1H-imidazol-1-yl)methyl]phenyl}prop-2-enoic acid	1-LOW	2	71.7749	2.0292	
5	3-(1H-imidazol-1-yl)-2-oxopropanoic acid	1-LOW	2	72.2215	−0.4632	
6	Imidazolepropionic Acid	1-LOW	2	79.7363	0.3578	
7	[1-hydroxy-2-(1H-imidazol-1-yl)-1-phosphonoethyl]phosphonic acid	1-LOW	2	9.6113	−1.1154	
1	2-benzyl-8-ethoxy-1,3-dimethyl-2H,4H-cyclohepta [c]pyrrol-4-one	1-LOW	1	40.2403	4.0653	PA2G5
1	2-methanesulfonyl-6-{1-methanesulfonyl-5H,6H,7H,8H,9H-cyclohepta [c]pyridin-3-yl}pyridine	1-LOW	1	66.3071	2.2195	SDHB
1	Isosorbide mononitrate	1-LOW	2	80.492	−1.2782	GSR

Note: Prediction level indicates the “credibility” of the prediction, with a higher numerical value indicating a higher level of credibility; average molecular ranking represents the average ranking of all targets hit by the ligand, with a smaller ranking value indicating a higher ranking among the hit targets; the higher the bioavailability score, the higher the bioavailability.

#### 3.4.2 Target-component fitting validation

**FIGURE 9:**
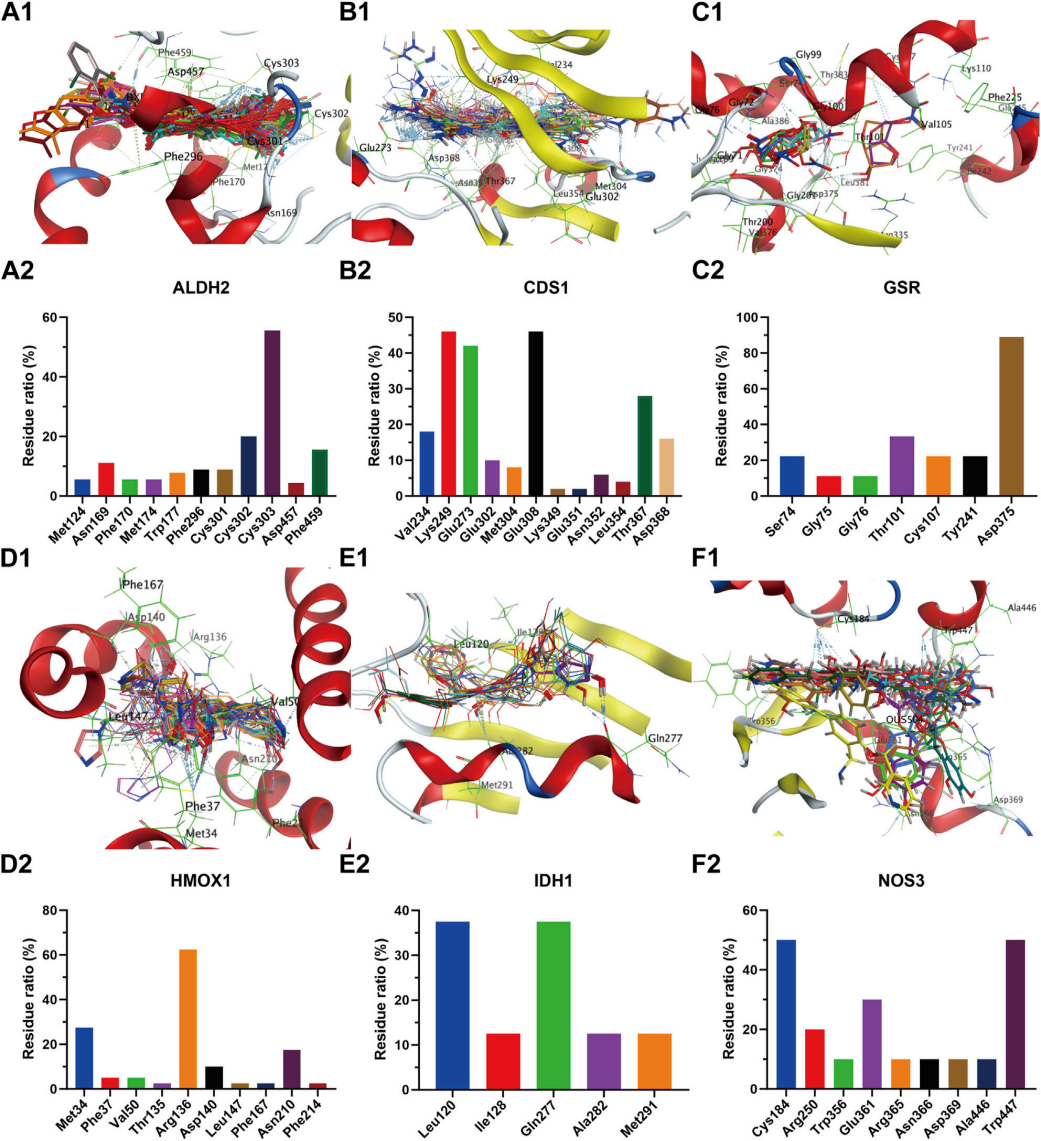
Compound overlay diagram in target (ALDH2, CDS1, GSR, HMOX1, IDH1, and NOS3)-component fitting (upper) and PLIF analysis chart (lower). The upper Figures **(A–F)** represent the overlay diagrams between the targets ALDH2, CDS1, GSR, HMOX1, IDH1, NOS3 and the components. The lower Figures **(A–F)** represent the PLIF fingerprint profiles of the interactions between the targets ALDH2, CDS1, GSR, HMOX1, IDH1, NOS3 and the components, respectively; the column height represents the proportion of compounds interacting with target residues.

**FIGURE 10:**
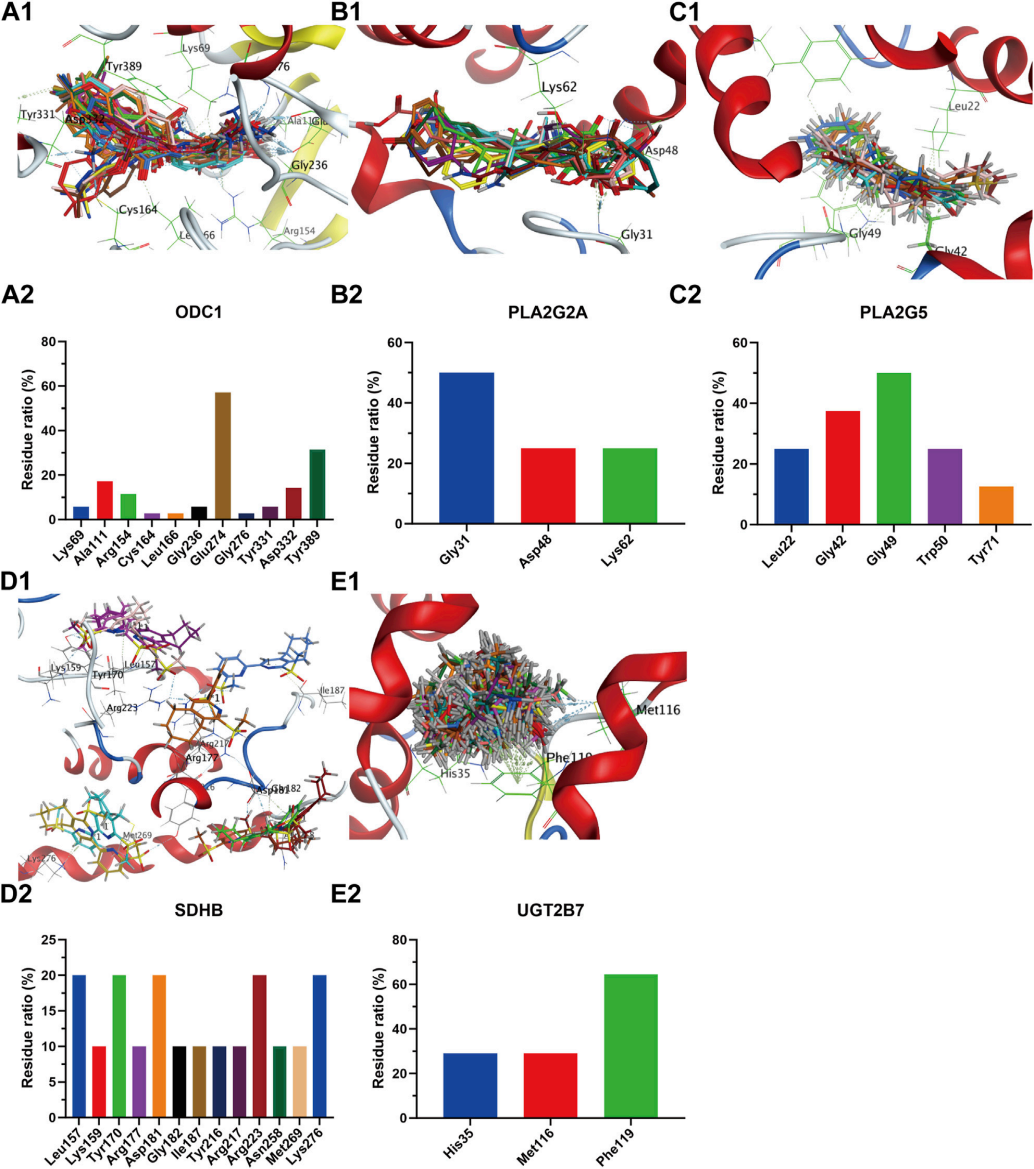
Compound overlay diagram in target (ODC1, PLA2G2A, PLA2G5, SDHB, UGT2B7)-component fitting (upper) and PLIF analysis chart (lower). The upper Figures **(A-E)** represent the overlay diagrams between the targets ODC1, PLA2G2A, PLA2G5, SDHB, UGT2B7 and the components. The lower Figures **(A-E)** represent the PLIF fingerprint profiles of the interactions between the targets ODC1, PLA2G2A, PLA2G5, SDHB, UGT2B7 and the components, respectively; the column height represents the proportion of compounds interacting with target residues.

The receptor proteins of ALDH2 (PDB code: 3INJ), CDS1 (PDB code: 2YIQ), ODC1 (PDB code: 7S3F), IDH1 (PDB code: 5DE1), NOS3 (PDB code: 6PP4), PLA2G2A (PDB code: 5G3N), and HMOX1 (PDB code: 6EHA) targets were downloaded from the PDB database (https://www.rcsb.org/), while the AlphaFold target receptor proteins of SDHB (P21912), UGT2B7 (P16662), PLA2G5 (P39877), and GSR (P00390) were downloaded from the UniProt database (https://www.uniprot.org/). Based on the reverse screening results of the lead compounds corresponding to each target in section 3.4.1, a corresponding compound database was established according to the method described in section 2.6.2. The active pockets were identified using the co-crystallized ligands of ALDH2 (BXB), CDS1 (YIQ), ODC1 (XAP), IDH1 (59D), NOS3 (OUS), PLA2G2A (X28), and HMOX1 (B5B) for ligand-target residue pocket fitting. The active pockets of UGT2B7, PLA2G5, and GSR targets were determined using the site finder function of the MOE docking module, and ligand-target residue pocket fitting was performed. A whole docking approach was adopted for ligand-target residue fitting for the SDHB target. The results of the ligand-target fitting scores are provided in Supplementary Table S2. The PLIF analysis results and 3D interaction diagrams of the ligand-target fitting residues are shown in Figures 9, 10.

As can be seen from the results presented in Supplementary Table S2, the mean docking scores of ALDH2, CDS1, ODC1, IDH1, NOS3, PLA2G2A, SDHB, UGT2B7, HMOX1, PLA2G5, and GSR with the corresponding database compounds ranged from −4.95 to −6.74, −5.55 to −7.91, −4.23 to −6.66, −6.93 to −8.91, −5.23 to −6.55, −6.04 to −7.33, −5.33, −5.40 to −5.93, −4.50 to −6.91, −6.74, and −5.37, respectively. As revealed by the PLIF and interaction analysis results of the target-residue docking in Figures 9, 10, Cys303, Cys302, and Phe459 were the primary residue binding sites for the target ALDH2; Glu308, Glu273, Lys249, and Val234 were the main residue binding sites for the target CDS1; Glu274 and Gly237 were the primary residue binding sites for the target ODC1; Ala282 and Tyr285 were the main residue binding sites for the target IDH1; Cys184, Trp356, and Trp447 were the primary residue binding sites for the target NOS3; Gly29, Gly31, and Lys62 were the main residue binding sites for the target PLA2G2A; Phe119, Met116, and His35 were the primary residue binding sites for the target UGT2B7; Arg136 and Met34 were the main residue binding sites for the target HMOX1; Gly49 and Gly42 were the primary residue binding sites for the target PLA2G5; whereas the residue binding sites for the targets SDHB and GSR were relatively dispersed.

## 4 Discussion

BPH is a prevalent urological disorder in males, particularly among elderly populations (Li S. et al., 2024). As a specific syndrome, BPH’s kidney insufficiency and blood stasis pattern exhibit complex pathological mechanisms and significant therapeutic challenges (Xu X. et al., 2022; Ma et al., 2023). Previous studies have demonstrated that multiple factors, including hormonal imbalance, increased oxidative stress, inflammatory responses, and alterations in various metabolic pathways, are closely associated with BPH progression (El et al., 2022; Wang et al., 2022; Inamura and Terada, 2024). TCM posits that kidney insufficiency and blood stasis constitute a critical pathogenic mechanism in BPH. Recent advancements in metabolomics, network pharmacology, and AI technologies have provided novel strategies for screening lead compounds in disease prevention and treatment (Ai et al., 2021; Wang et al., 2023; Chacko et al., 2022). This study established a metabolite-reaction-enzyme-gene network for kidney insufficiency and blood stasis-pattern BPH by integrating clinical metabolomics and network pharmacology with an AI reverse-screening platform to identify potential bioactive compounds targeting disease-specific pathways. Serum biochemical analyses revealed that compared to the healthy group, the BPH group exhibited hormonal disturbances characterized by decreased serum testosterone (T), elevated estradiol (E2), and a reduced T/E2 ratio. Age-related declines in T levels and increases in E2 levels contribute to T/E2 ratio imbalance, a metabolic dysfunction strongly linked to prostatic hyperplasia (Xu et al., 2016). SRD5α2 is a key enzyme in androgen metabolism, which converts Testosterone (T) to the more active Dihydrotestosterone (DHT) in the prostate. DHT plays a central role in the pathogenesis of benign prostatic hyperplasia (BPH) by continuously activating the androgen receptor signaling pathway and promoting the proliferation of prostate epithelial cells (Wang et al., 2017). Therapeutic strategies employing 5α-reductase inhibitors to suppress testosterone conversion to dihydrotestosterone (DHT), thereby reducing intraprostatic DHT levels, or estrogen receptor antagonists to modulate systemic T/E2 ratios, have proven effective in alleviating BPH symptoms (Ishikawa et al., 2006). This is consistent with the increase of SRD5α2, the imbalance of T and E ratio in the BPH group in the present study, and the endocrine hormone disorder in the pathogenesis of BPH. In this study, elevated levels of NF-κB p65 and TGF-β in the BPH group aligned with established mechanisms of chronic inflammation in BPH pathogenesis (Inamura and Terada, 2024; He et al., 2016).

Compared with the control group, the serum metabolic profile of BPH patients is significantly abnormal, involving multiple pathophysiological pathways such as amino acid metabolism, energy metabolism, oxidative stress, steroid hormones and phospholipid remodelling. The levels of glutamic acid, pyroglutamic acid and its derived dipeptides (such as glutamylvaline and leucyl-glutamate) increased, indicating the disorder of glutamyl metabolism and the enhancement of oxidative stress (Yang et al., 1995). Glutamic acid may activate the MAPK/ERK pathway through glutamate receptors (such as mGluR) in prostate stromal cells and promote cell proliferation (Koda et al., 2023). The accumulation of TCA cycle intermediates, such as oxoglutaric and citric acid, reflects cell mitochondrial dysfunction (Desideri et al., 2015; Martínez-Reyes and Chandel, 2020). However, the elevation of long-chain hydroxyfatty acids, such as 3-hydroxydodecanedioic acid, is associated with impaired mitochondrial β-oxidation (Guerra et al., 2022; Ribas and Vargas, 2022). The decrease of keto acids (such as levulinic acid, 3-oxohexadecanoic acid) indicates the disorder of peroxisome metabolism, which affects the regular operation of the α-oxidation function (Pflanz et al., 2019). Mitochondrial dysfunction may increase the reactive oxygen species (ROS) generation, activate the NF-κ B pro-inflammatory pathways, and drive prostate fibrosis (Adrian et al., 2024). BPH patients also show significant oxidative stress characteristics, as shown by increased levels of 4-hydroxynonenal (4-HNE), 9, 10-Dhome and xanthosine (Poli and Schaur, 2000; Chacko et al., 2016; Chen et al., 2022). At the same time, the levels of antioxidant substances (such as bilirubin and sphingosine 1-phosphate) are decreased (Hubáček and Vítek, 2019; Wollny et al., 2022), and the above changes are closely related to chronic low-grade inflammation. The accumulation of pro-inflammatory mediators such as 6-trans-12-epi-LTB4 and bicyclo PGE2 promotes matrix proliferation through the TGF-β/Smad pathway (Kamata et al., 2019; Abo-El Fetoh et al., 2023). Regarding steroid metabolism, increased pregnenolone levels suggest enhanced steroid synthesis, while increased stanolone glucuronate is closely related to increased local dihydrotestosterone (DHT) activity (Witzig et al., 2020; Choi et al., 2003). In addition, parallel metabolic pathways exist between sulfated and unconjugated steroids, and the synthesis of testosterone is mainly dependent on sulfated precursors, which maintain the balance of steroid hormones under the regulation of steroid sulfatase (Sánchez-Guijo et al., 2016; Saez et al., 1967). The decreased level of testosterone sulfate is closely related to the abnormal regulation of the 5α-reductase-DHT axis, and DHT stimulates the proliferation of prostate cells through the androgen receptor signalling pathway (Cooper and Page, 2014). Abnormal phospholipid metabolism with increased lysophosphatides (LysoPC, LysoPA) and decreased phospholipids of long-chain polyunsaturated fatty acids (such as PC(22:0/22:6), PE (22:5/22:6)) suggests membrane phospholipid degradation or activation of pro-proliferation signals (Koeberle et al., 2012; Hofmanová et al., 2021). The metabolic imbalance of oxidative stress-inflammation-hormones in BPH provides a potential molecular basis for targeted interventions, such as anti-oxidation, anti-inflammation and hormonal regulation.

Further metabolomics enrichment studies have shown that BPH involves dysregulation of multiple metabolic pathways, including the tricarboxylic acid cycle, glycerophospholipid metabolism, porphyrin metabolism, uric acid cycle, and amino acid metabolism (Yu et al., 2021; Wang S. et al., 2024). Metabolomic analysis (Figures 5, 6) revealed glycerophospholipid, alanine-aspartate-glutamate, arginine biosynthesis, glutathione, TCA cycle, and porphyrin metabolism as critical endogenous regulatory pathways in BPH, demonstrating significant crosstalk. The TCA cycle, a central pathway in cellular energy metabolism (Martínez-Reyes and Chandel, 2020), undergoes metabolic reprogramming in hyperplastic prostatic tissues to meet the energy demands of rapidly proliferating cells. Citrate and α-ketoglutarate (α-KG), key TCA cycle intermediates, play pivotal roles in regulating cellular energy metabolism. Notably, citrate—a significant component of prostatic fluid—is markedly elevated in hyperplastic prostates due to glandular epithelial hyperplasia and ductal dilation (Cooper and Farid, 1963; Kanazawa et al., 1964). Beyond its canonical role in oxidative decarboxylation via isocitrate dehydrogenase (IDH1) and succinate dehydrogenase (SDHB), α-KG modulates redox homeostasis and biosynthetic processes by influencing diverse metabolic pathways. Specifically, α-KG regulates cell proliferation, apoptosis, and differentiation through metabolic reprogramming and hormonal signalling, directly implicating it in BPH pathogenesis (Kang et al., 2020; Xu C. et al., 2022). Dysregulation of the TCA cycle further exacerbates intracellular oxidative stress, accelerating BPH progression (Wang et al., 2022; Mailloux et al., 2007). Glycerophospholipids, the most abundant in biological systems, are structural components of cell membranes and mediators of cellular signalling and protein recognition (Wang Y. et al., 2024). Aberrant glycerophospholipid metabolism significantly contributes to BPH pathophysiology (Geng et al., 2014), with dysregulated levels of phosphatidylethanolamine (PE) and phosphatidylcholine (PC) closely linked to disease initiation and progression (Fan et al., 2024). Patients with BPH and histologically confirmed chronic inflammation exhibit distinct metabolic profiles, underscoring the critical role of glycerophospholipid metabolism in inflammation-driven prostatic hyperplasia (Li J. et al., 2024). Phospholipase A2 (PLA2), a key enzyme in glycerophospholipid catabolism, hydrolyzes glycerophospholipids to release bioactive mediators such as arachidonic acid, thereby modulating inflammatory responses and cellular proliferation in BPH (Hayashi et al., 2022; Turnaev et al., 2022). Additional contributors to prostatic disease progression include ALDH2 and CDS1, which are implicated in glycerophospholipid-related pathways (Seok et al., 2013; Ward et al., 2021; Cui et al., 2023). Porphyrin metabolism is functionally linked to BPH through heme oxygenase-1 (HO-1), which is overexpressed in hyperplastic prostates. HO-1 catalyzes heme degradation into biliverdin, a process potentially involved in the pathogenesis of both BPH and prostate cancer (Maines and Abrahamsson, 1996). Similarly, UGT2B, a porphyrin metabolism-associated enzyme, influences BPH progression via androgen metabolism regulation (Zhan et al., 2022). Uric acid and amino acid cycling further participate in prostatic disease mechanisms (Morris, 2002; Afify et al., 2020). Importantly, α-ketoglutarate (α-KG) and glutamate serve as pivotal metabolites within the interconnected pathways of alanine-aspartate-glutamate metabolism, arginine biosynthesis, glutathione metabolism, and the tricarboxylic acid (TCA) cycle, thereby functioning as central nodes that integrate these metabolic processes (Ren et al., 2023).

The integrated metabolomic and network pharmacological findings (Figure 7) demonstrate that targets including ALDH2 and PLA2 participate in glycerophospholipid metabolism by regulating metabolites such as PC and PE; targets including IDH1 and SDHB are involved in the TCA cycle through metabolites like citrate and 2-oxoglutarate; targets including ALDH2 and NOS3 modulate uric acid cycling and amino acid metabolism via metabolites including 2-oxoglutarate, glutamate, and 5-oxoproline. Serum biochemical analyses revealed that the BPH group exhibited hormonal dysregulation (e.g., reduced T/E2 ratio), elevated transforming growth factor levels, and endogenous metabolic perturbations. These results are consistent with the target enrichment profiles of network pharmacology and previously reported pathological mechanisms in BPH (Li J. et al., 2024; Li et al., 2019; Jing, 2021). Consistent with this, the results of sub-network mining of PPI interaction of network pharmacological targets showed that the sub-networks mainly included the overlapping integrated targets as HMOX1, UGT2B7, GCLC and GSTM1 (sub-network 1, Figure 8A2), ALDH2 and GSTP1 (sub-network 2, Figure 8A3), and PLA2G2A, PLA2G5, GSR, NOS3, SDHB (subnetwork 3, Figure 8A4). The results of enrichment analysis showed that the core targets, such as UGT2B/GST families (chemoresistance/detoxification), mitochondrial regulators (IDH1/SDHB/SDHD; TCA cycle/metabolic-epigenetic crosstalk), PLA2G2A/PLA2G5 (lipid-inflammatory signaling via arachidonic acid metabolism), ALDH2 (aldehyde detoxification/redox balance), HMOX1 (heme degradation/oxidative stress resolution), and NOS3 (NO-mediated vascular homeostasis) converge on a multidimensional network spanning glutathione/phospholipid metabolism (GO), chemical carcinogenesis (KEGG), and drug resistance pathways. These targets synergistically regulate detoxification-oxidative stress-lipid signaling axes, linking mitochondrial dysfunction, inflammation, and metabolic syndrome comorbidities. Reverse screening was performed using the Yaozh Database-Natural Product AI Engine Platform and its proprietary algorithms, through which 49 potential ligand-target interaction compounds were identified from 11 clinically druggable targets derived from integrated metabolomics and network pharmacology analysis. According to the results (Table 1; Figures 9, 10), in addition to SHDB, GSR, and PLA2G5, the predicted validity levels for targets such as ALDH2, CDS1, ODC1, IDH1, NOS3, UGT2B7, and HMOX-1 are relatively high. Moreover, during the ligand-target docking process, the ligand conformations exhibit close superposition, and there are prominent features of ligand affinity with clustered target residues.

BPH is a common urinary system disease in males, characterized pathologically by an enlarged prostate gland that compresses the urethra, leading to symptoms such as frequent urination, urgent urination, difficult urination, and urinary retention (Li S. et al., 2024). Contemporary medical treatment primarily involves the use of 5-α reductase inhibitors, α-blockers, and M-receptor antagonists for symptomatic relief; however, most of these treatments have limited efficacy and may cause varying degrees of toxic and side effects (Strand et al., 2017; Sun et al., 2023; Lei and Ling, 2017). BPH aligns with the descriptions of “Jīng Lóng” and “Lóng Bì” in TCM, where constitutional weakness and deficiency of the liver and kidney are common internal pathogenic factors for BPH (Mao et al., 2021). The Foot-Jueyin Liver Meridian can course through the urinary and reproductive systems, maintaining normal physiological functions. Deficiency of the liver and kidney and abnormal spleen and stomach transportation leads to a lack of qi and blood in the liver, further causing the accumulation of damp-heat or phlegm-dampness, resulting in qi stagnation and blood stasis or meridian obstruction. Clinically, this manifests as weakness and soreness of the knees, stagnation pain, and urodynamic disorders related to the kidney and bladder meridians, among other kidney and liver meridian pathologies (He et al., 2023). In this study, BPH exhibited metabolic disorders such as those in the TCA cycle, glycerophospholipid metabolism, uric acid and amino acid cycles. It is consistent with understanding abnormal qi and blood production in the liver as described in TCM for BPH. Further integrative metabolomics-network pharmacology analysis revealed that these differential metabolic pathways can directly regulate BPH through metabolite or metabolite-reaction-enzyme-gene networks or intervene in the progression of BPH by regulating inflammation, oxidative stress, fibrosis, and other pathways.

Based on clinical practice, this study linked the metabolic phenotypes, molecular targets, signalling pathways, and potential therapeutic compounds of diseases. This research idea of combining modern precision medicine with the holistic view of traditional Chinese medicine is a concrete embodiment of the research paradigm of biological systems medicine. Compared with the traditional animal experimental model, the content of this study is closer to the clinical real world. Through the integrated analysis of metabolomics and network pharmacology on the research system, the macro-regulation at the system biology level and the accurate positioning of molecular targets are realised. On this basis, the target-based reverse screening of compounds was combined with ligand-target interaction fitting to obtain potential therapeutic compounds with clear structure-activity relationships. This reverse research paradigm from clinical phenotypia-molecular mechanism-therapeutic drugs significantly improves the clinical translation potential of candidate drugs. Thus, the results of this study provide valuable insights into targeted therapy and drug development for BPH, have the potential to improve patient outcomes through personalised treatment strategies, and provide an innovative platform for the development of evidence-based TCM treatment options for BPH and other complex diseases (Li et al., 2023; Zhang, 2023). In this study, only the molecular docking technology was used to verify the affinity of the selected compounds with the corresponding targets and the position of amino acid residues. The animal model of BPH should be used to verify the targets and potential compounds that were obtained in the future. Furthermore, this study identified aberrant levels of lipid constituents, specifically phosphatidylethanolamine and phosphatidylcholine, in the disease state. Consequently, it is imperative to employ more precise lipid metabolomics techniques to elucidate the alterations and associated regulatory mechanisms of PE/PC.

## 5 Conclusion

This study successfully integrated clinical metabolomics and network pharmacology to identify key therapeutic targets and potential compounds for treating kidney insufficiency and blood stasis-type BPH. The findings revealed a hormonal imbalance and elevated levels of inflammatory and fibrotic markers in BPH patients. These were associated with significant alterations in metabolic pathways such as glycerophospholipid metabolism, the TCA cycle, glutathione metabolism, and porphyrin metabolism. The combined approach led to identifying 178 potential BPH targets and constructing compound-reaction-enzyme-gene networks, which were further refined to 23 core targets. Reverse screening and target-component validation confirmed the high predictive reliability of seven core targets, including ALDH2, CDS1, ODC1, IDH1, NOS3, UGT2B7, and HMOX-1, as evidenced by the tight ligand conformation overlap and prominent ligand affinity-driven target residue clustering. These results offer valuable insights for developing targeted therapies and advancing drug discovery for BPH, potentially improving patient outcomes through personalised treatment strategies.

## Data Availability

The original contributions presented in the study are publicly available. This data can be found here: https://www.metabolomicsworkbench.org, where it has been assigned Study ID ST003986.
