# Supplementary material for: Unveiling therapeutic targets and preventive components for kidney insufficiency and blood stasis-type BPH: bridging metabolomics, network pharmacology and reverse screening
Source: Front Pharmacol. 2025 Jun 19;16:1584766. doi: 10.3389/fphar.2025.1584766 (PMC12230579; doi:10.3389/fphar.2025.1584766)
Supplement: Supplementary file 1 [file Supplementaryfile1.docx]

Supplementary Material


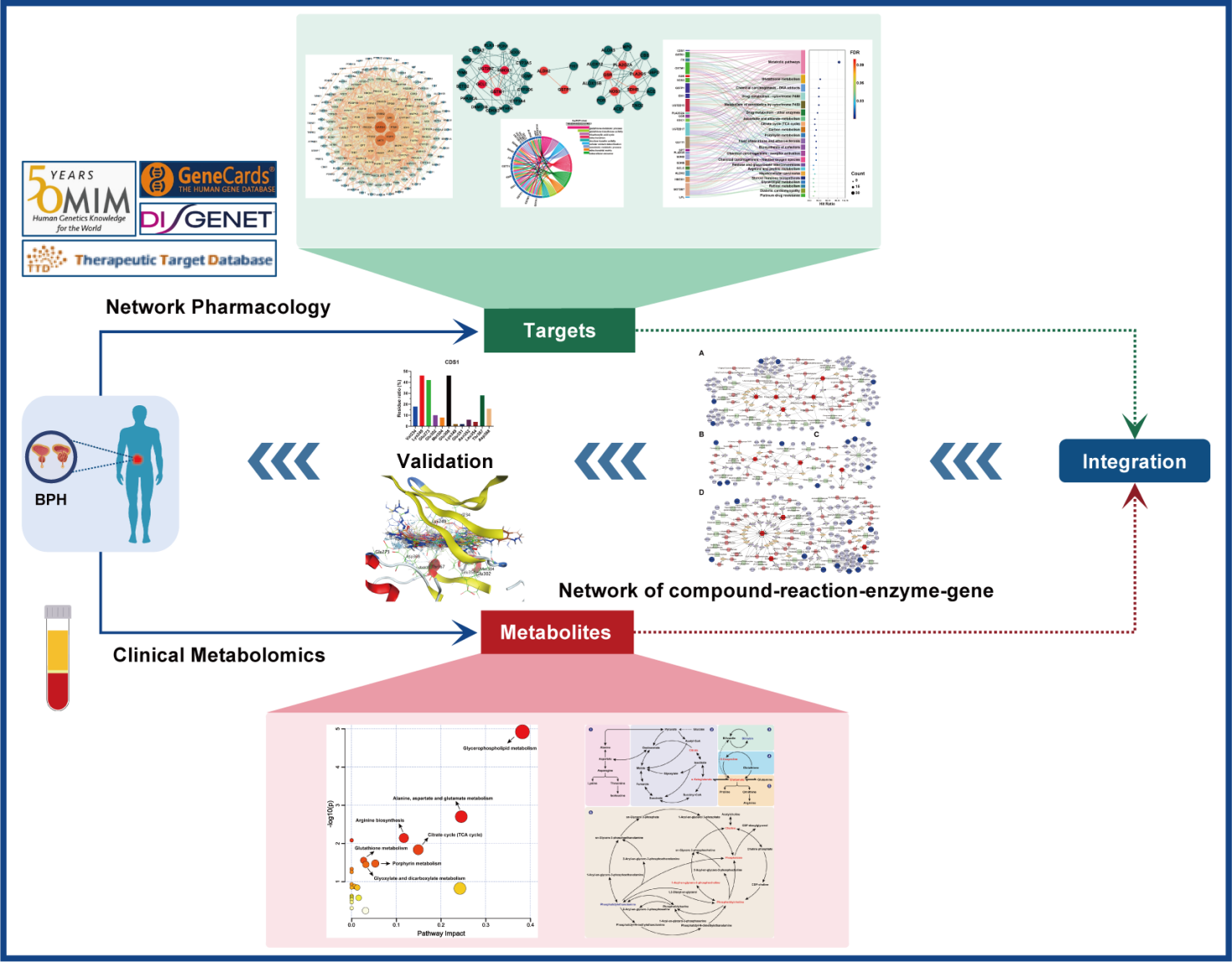


**Supplementary Figure 1.** The research flow chart.


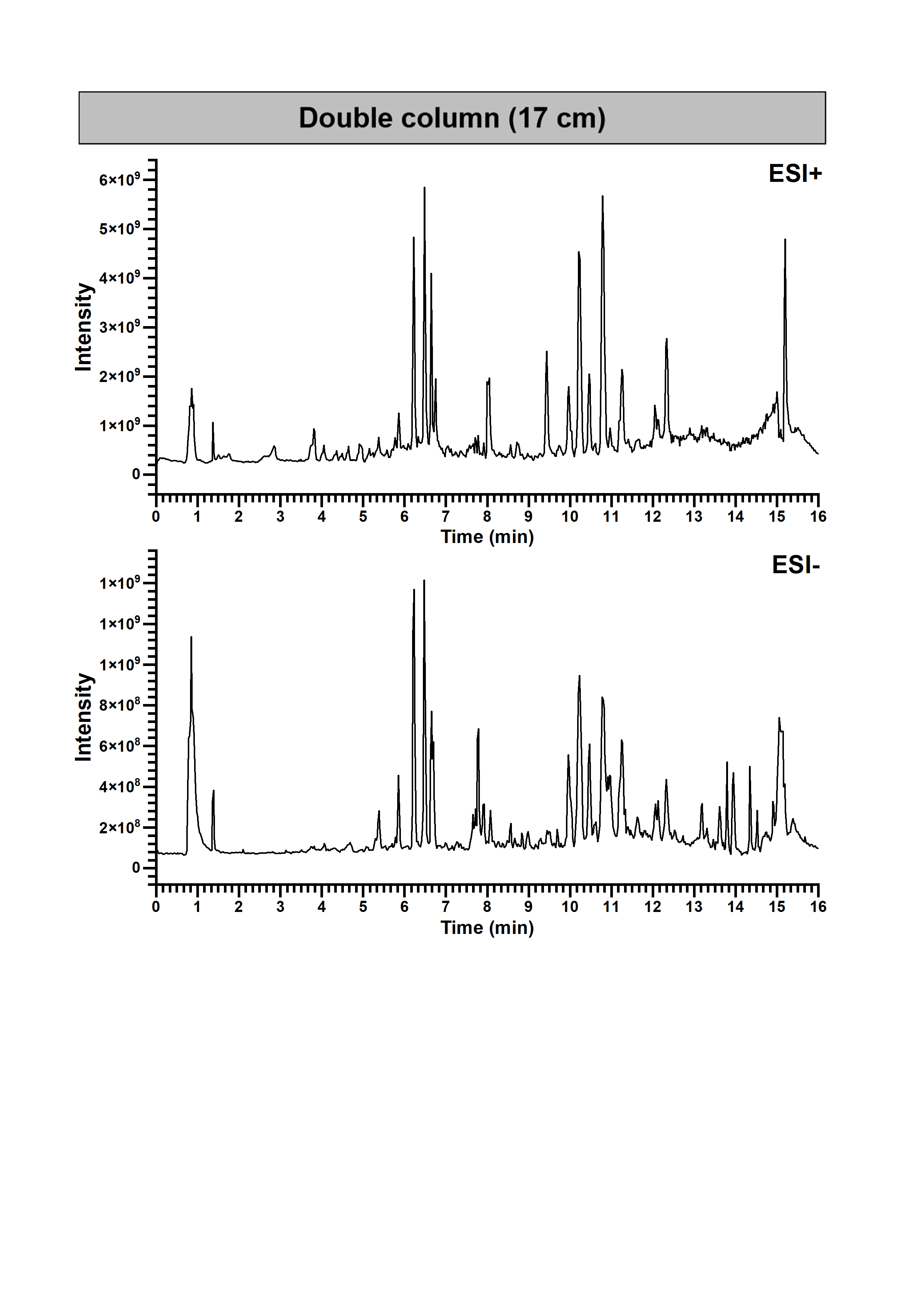


**Supplementary Figure 2.** Total ion chromatograms of serum samples under positive ion mode and negative ion mode. The upper Figure shows the total ion chromatogram in positive ion mode, while the below Figure shows the total ion chromatogram in negative ion mode.

**Supplementary Table 1.** Differential Serum Metabolites Distinguishing BPH Patients from Healthy Individuals

| **No.** | **HMDB ID** | **Metabolites Name** | **Formula** | **tR/min** | **Adduct Ion** | **M/Z** | **BPH vs. Control** |
| --- | --- | --- | --- | --- | --- | --- | --- |
| 1 | HMDB0000097 | Choline | C_5_H_13_NO | 0.871 | [M+H]^+^ | 104.10724 | ↑**** |
| 2 | HMDB0000062 | Carnitine | C_7_H_15_NO_3_ | 0.886 | [M+H]^+^ | 162.11231 | ↓** |
| 3 | HMDB0000230 | N-Acetylneuraminic acid | C_11_H_19_NO_9_ | 0.887 | [M-H]^-^ | 308.09906 | ↑**** |
| 4 | HMDB0000148 | Glutamic acid | C_5_H_9_NO_4_ | 0.893 | [M+H]^+^ | 148.06031 | ↑**** |
| 5 | HMDB0000208 | Oxoglutaric acid | C_5_H_6_O_5_ | 0.905 | [M+FA-H]^-^ | 191.01893 | ↑**** |
| 6 | HMDB0000094 | Citric acid | C_6_H_8_O_7_ | 1.382 | [M-H]^-^ | 191.01886 | ↑**** |
| 7 | HMDB0000267 | Pyroglutamic acid | C_5_H_7_NO_3_ | 1.413 | [M+H]^+^ | 130.04984 | ↑* |
| 8 | HMDB0028832 | Glutamylvaline | C_10_H_18_N_2_O_5_ | 2.517 | [M-H]^-^ | 245.11431 | ↑**** |
| 9 | HMDB0000720 | Levulinic acid | C_5_H_8_O_3_ | 2.608 | [M-H]^-^ | 115.03862 | ↓*** |
| 10 | HMDB0000325 | 3-Hydroxysuberic acid | C_8_H_14_O_5_ | 3.486 | [M-H]^-^ | 189.07604 | ↑**** |
| 11 | HMDB0028928 | Leucyl-Glutamate | C_11_H_20_N_2_O_5_ | 3.779 | [M+H]^+^ | 261.14405 | ↑**** |
| 12 | HMDB0028823 | Glutamylleucine | C_11_H_20_N_2_O_5_ | 3.941 | [M-H]^-^ | 259.1301 | ↑**** |
| 13 | HMDB0013267 | N-Decanoylglycine | C_12_H_23_NO_3_ | 4.331 | [M+Na]^+^ | 252.15661 | ↓**** |
| 14 | HMDB0060054 | bicyclo-PGE2 | C_20_H_30_O_4_ | 4.624 | [M-H]^-^ | 315.19278 | ↑* |
| 15 | HMDB0009780 | PI(16:0/16:2(9Z,12Z)) | C_41_H_75_O_13_P | 5.658 | [M+H]^+^ | 807.4979 | ↓**** |
| 16 | HMDB0040133 | Pectenotoxin 2 secoacid | C_47_H_72_O_15_ | 5.763 | [M+2H]^+^ | 439.24985 | ↓**** |
| 17 | HMDB0008728 | PC(22:6/18:1) | C_48_H_82_NO_8_P | 5.776 | [M+2H]^+^ | 416.79704 | ↑**** |
| 18 | HMDB0009639 | PE(22:5/22:6) | C_49_H_76_NO_8_P | 6.248 | [M+2H]^+^ | 419.77448 | ↓**** |
| 19 | HMDB0000413 | 3-Hydroxydodecanedioic acid | C_12_H_22_O_5_ | 6.722 | [M-H]^-^ | 245.13931 | ↑**** |
| 20 | HMDB0000784 | Azelaic acid | C_9_H_16_O_4_ | 7.242 | [M-H]^-^ | 187.09673 | ↑**** |
| 21 | HMDB0008551 | PC(22:0/22:6) | C_52_H_94_NO_7_P | 7.595 | [M+2H]^+^ | 438.84786 | ↓** |
| 22 | HMDB0003252 | Thromboxane B2 | C_20_H_34_O_6_ | 7.63 | [M-H]^-^ | 369.22858 | ↓**** |
| 23 | HMDB0000054 | Bilirubin | C_33_H_36_N_4_O_6_ | 7.674 | [M+H]^+^ | 585.27003 | ↓**** |
| 24 | HMDB0002833 | Testosterone sulfate | C_19_H_28_O_5_S | 7.776 | [M-H]^-^ | 367.15865 | ↓**** |
| 25 | HMDB0030993 | 2-Carboxy-4-tridecanolide | C_14_H_24_O_4_ | 7.924 | [M+H]^+^ | 257.1742 | ↑**** |
| 26 | HMDB0000394 | 3-Hydroxytetradecanedioic acid | C_14_H_26_O_5_ | 7.927 | [M+H-H_2_O]^+^ | 257.17425 | ↑*** |
| 27 | HMDB0000623 | Dodecanedioic acid | C_12_H_22_O_4_ | 7.987 | [M-H]^-^ | 229.14426 | ↑* |
| 28 | HMDB0028945 | Lysylarginine | C_12_H_26_N_6_O_3_ | 8.716 | [M-H+HAc]^-^ | 301.20235 | ↑** |
| 29 | HMDB0000277 | Sphingosine 1-phosphate | C_18_H_38_NO_5_P | 9.174 | [M+H]^+^ | 380.25546 | ↓**** |
| 30 | HMDB0005088 | 6-trans-12-epi-LTB4 | C_20_H_32_O_4_ | 9.361 | [M-H]^-^ | 335.22331 | ↑*** |
| 31 | HMDB0031094 | Glycerol tributanoate | C_15_H_26_O_6_ | 9.388 | [M-H]^-^ | 301.16599 | ↑**** |
| 32 | HMDB0031885 | 6-Hydroxypentadecanedioate | C_15_H_28_O_5_ | 9.415 | [M-H]^-^ | 287.18679 | ↑**** |
| 33 | HMDB0032783 | Porrigenin A | C_27_H_44_O_5_ | 9.668 | [M+H]^+^ | 449.32637 | ↑**** |
| 34 | HMDB0034658 | 9-Acetoxyfukinanolide | C_17_H_24_O_4_ | 10.006 | [M-H]^-^ | 291.16054 | ↑** |
| 35 | HMDB0006227 | 1,24,25-Trihydroxyvitamin D2 | C_28_H_44_O_4_ | 10.429 | [M+ACN+Na]^+^ | 508.33911 | ↑*** |
| 36 | HMDB0010381 | LysoPC(15:0/0:0) | C_23_H_48_NO_7_P | 10.465 | [M-H+HAc]^-^ | 540.33127 | ↑** |
| 37 | HMDB0004704 | 9,10-DHOME | C_18_H_34_O_4_ | 11.127 | [M-H]^-^ | 295.22805 | ↑** |
| 38 | HMDB0062316 | PA(18:3(6Z,9Z,12Z)/0:0) | C_21_H_37_O_7_P | 11.209 | [M-H]^-^ | 431.22064 | ↑**** |
| 39 | HMDB0010391 | LysoPC(20:1(11Z)/0:0) | C_28_H_56_NO_7_P | 11.235 | [M+H]^+^ | 550.39084 | ↑**** |
| 40 | HMDB0062308 | PA(20:5(5Z,8Z,11Z,14Z,17Z)/0:0) | C_23_H_37_O_7_P | 11.304 | [M-H]^-^ | 455.22107 | ↑**** |
| 41 | HMDB0002157 | 27-Norcholestanehexol | C_26_H_46_O_6_ | 11.351 | [M-H]^-^ | 453.32292 | ↓*** |
| 42 | HMDB0013122 | LysoPC(P-18:0/0:0) | C_26_H_54_NO_6_P | 11.627 | [M+H]^+^ | 508.37557 | ↑*** |
| 43 | HMDB0010392 | LysoPC(20:2(11Z,14Z)/0:0) | C_28_H_54_NO_7_P | 11.662 | [M+H]^+^ | 548.37033 | ↑*** |
| 44 | HMDB0006203 | Stanolone glucuronate | C_25_H_38_O_8_ | 11.902 | [M+H]^+^ | 467.26081 | ↑*** |
| 45 | HMDB0013058 | S-(9-Deoxy-δ9,12-PGD2)-glutathione | C_30_H_47_N_3_O_10_S | 12.059 | [M-H]^-^ | 640.29328 | ↑*** |
| 46 | HMDB0010384 | LysoPC(18:0/0:0) | C_26_H_54_NO_7_P | 12.062 | [M+H]^+^ | 524.37035 | ↑*** |
| 47 | HMDB0004362 | 4-Hydroxynonenal | C_9_H_16_O_2_ | 12.109 | [2M+FA-H]^-^ | 357.22874 | ↑**** |
| 48 | HMDB0007852 | LysoPA(0:0/18:2(9Z,12Z)) | C_21_H_39_O_7_P | 12.402 | [M-H]^-^ | 433.23652 | ↑**** |
| 49 | HMDB0034079 | Homodolichosterone | C_29_H_48_O_5_ | 12.492 | [M+H]^+^ | 477.3581 | ↑**** |
| 50 | HMDB0033786 | Plastoquinone 3 | C_23_H_32_O_2_ | 12.9 | [M+H]^+^ | 341.24692 | ↑**** |
| 51 | HMDB0001358 | Retinal | C_20_H_28_O | 12.91 | [M+H+MeOH]^+^ | 317.24696 | ↑**** |
| 52 | HMDB0000253 | Pregnenolone | C_21_H_32_O_2_ | 12.911 | [M+H]^+^ | 317.24701 | ↑**** |
| 53 | HMDB0031678 | Pipericine | C_22_H_41_NO | 12.922 | [M+H]^+^ | 336.32536 | ↓* |
| 54 | HMDB0000299 | Xanthosine | C_10_H_12_N_4_O_6_ | 12.941 | [M-H]^-^ | 283.07058 | ↑**** |
| 55 | HMDB0002259 | Heptadecanoic acid | C_17_H_34_O_2_ | 12.95 | [M+H]^+^ | 293.24683 | ↑**** |
| 56 | HMDB0000476 | 3-Oxo-4,6-choladienoic acid | C_24_H_34_O_3_ | 13.001 | [M-H]^-^ | 369.24379 | ↑**** |
| 57 | HMDB0006294 | 16-Hydroxyhexadecanoic acid | C_16_H_32_O_3_ | 13.282 | [M-H]^-^ | 271.22813 | ↑* |
| 58 | HMDB0010733 | 3-Oxohexadecanoic acid | C_16_H_30_O_3_ | 14.351 | [2M-H]^-^ | 539.43235 | ↓**** |

The symbol “↑” indicates an increase in the normalized peak area for the BPH group, while the symbol “↓” indicates a decrease in the normalized peak area for the BPH group.

**Supplementary Table 2.** Fitness scores of potential compounds docking with phenotype-regulated targets

| **No.** | **Compounds Name** | **Targets** | **Scores** |
| --- | --- | --- | --- |
| 1 | 2-[(4-methyl-2-oxo-2H-chromen-7-yl)oxy]acetonitrile | ALDH2 | -5.86 ± 0.13 |
| 2 | Prunetin | ALDH2 | -6.72 ± 0.56 |
| 3 | methyl 2-[(4-methyl-2-oxo-2H-chromen-7-yl)oxy]propanoate | ALDH2 | -6.74 ± 0.2 |
| 4 | 14,15-dimethyl-9,13-dioxatetracyclo[8.7.0.0²,⁷.0¹²,¹⁶]heptadeca-1(17),2(7),3,5,10,12(16),14-heptaen-8-one | ALDH2 | -5.45 ± 0.58 |
| 5 | 13,14-dimethyl-8,12-dioxatetracyclo[7.7.0.0²,⁶.0¹¹,¹⁵]hexadeca-1(16),2(6),9,11(15),13-pentaen-7-one | ALDH2 | -5.4 ± 0.49 |
| 6 | 3,4,8,9-tetramethyl-7H-furo[2,3-f]chromen-7-one | ALDH2 | -4.95 ± 0.44 |
| 7 | 2,3,5,6-tetramethyl-7H-furo[3,2-g]chromen-7-one | ALDH2 | -5.99 ± 0.31 |
| 8 | 3,5-dimethyl-6-propyl-7H-furo[3,2-g]chromen-7-one | ALDH2 | -6.38 ± 0.31 |
| 9 | 14,15-dimethyl-9,13-dioxatetracyclo[8.7.0.0²,⁷.0¹²,¹⁶]heptadeca-1(17),2(7),10,12(16),14-pentaen-8-one | ALDH2 | -5.41 ± 0.4 |
| 10 | 2,3-dimethyl-5-propyl-7H-furo[3,2-g]chromen-7-one | ALDH2 | -5.53 ± 0.27 |
| 11 | 6-ethyl-2,3,5-trimethyl-7H-furo[3,2-g]chromen-7-one | ALDH2 | -6.32 ± 0.19 |
| 12 | 2,3,5-trimethyl-6-(propan-2-yl)-7H-furo[3,2-g]chromen-7-one | ALDH2 | -6.36 ± 0.28 |
| 13 | 15,16-dimethyl-10,14-dioxatetracyclo[9.7.0.0²,⁸.0¹³,¹⁷]octadeca-1(18),2(8),11,13(17),15-pentaen-9-one | ALDH2 | -5.16 ± 0.47 |
| 14 | 5-butyl-2,3-dimethyl-7H-furo[3,2-g]chromen-7-one | ALDH2 | -5.72 ± 0.3 |
| 15 | N-(1,3-benzodioxol-5-ylmethyl)-2,6-dichlorobenzamide (BXB: Co-crystallized ligand of 3INJ) | ALDH2 | -6.28 ± 0.33 |
| 1 | 2-Bromo-4-(5-Hydroxy-2-Imino-3H-Imidazol-4-Ylidene)-1H,5H,6H,7H-Pyrrolo[2,3-C]Azepin-8-One | CDS1 | -5.97 ± 0.22 |
| 2 | 2-Bromo-4-[(4Z)-5-Hydroxy-2-Imino-3H-Imidazol-4-Ylidene]-1H,5H,6H,7H-Pyrrolo[2,3-C]Azepin-8-One | CDS1 | -5.97 ± 0.33 |
| 3 | 2-Imino-5-{8-Oxo-1H,5H,6H,7H-Pyrrolo[2,3-C]Azepin-4-Ylidene}Imidazolidin-4-One | CDS1 | -5.72 ± 0.29 |
| 4 | 2-Bromo-4-[(4E)-5-Hydroxy-2-Imino-3H-Imidazol-4-Ylidene]-1H,5H,6H,7H-Pyrrolo[2,3-C]Azepin-8-One | CDS1 | -5.85 ± 0.18 |
| 5 | 5-{3-Bromo-8-Oxo-1H,5H,6H,7H-Pyrrolo[2,3-C]Azepin-4-Ylidene}-2-Iminoimidazolidin-4-One | CDS1 | -5.55 ± 0.18 |
| 6 | 2-Bromo-4-[(4Z)-2,5-Dihydroxyimidazol-4-Ylidene]-1H,5H,6H,7H-Pyrrolo[2,3-C]Azepin-8-One | CDS1 | -5.99 ± 0.2 |
| 7 | (E)-5-(1-(2-Carbamimidoylhydrazono)ethyl)-N-(1H-Indol-6-YL)-1H-Indole-2-Carboxamide (YIQ: Co-crystallized ligand of 2YIQ) | CDS1 | -7.91 ± 0.25 |
| 1 | 8,8-Dimethyl-3-(2,4,5-Trimethoxyphenyl)-2H,3H-Pyrano[2,3-F]Chromen-4-One | ODC1 | -6.66 ± 0.3 |
| 2 | Pongachalcone Ii | ODC1 | -6.52 ± 0.27 |
| 3 | Isobavachromene | ODC1 | -6.11 ± 0.18 |
| 4 | 5-Methoxy-2,2-dimethyl-7-[2-(4-hydroxyphenyl)ethenyl]-2H-1-benzopyran | ODC1 | -5.86 ± 0.16 |
| 5 | 5-Methoxy-2,2-dimethyl-7-[2-(4-hydroxy-3-methoxyphenyl)ethenyl]-2H-1-benzopyran | ODC1 | -6.16 ± 0.17 |
| 6 | 3-Aminooxy-1-Aminopropane (XAP: Co-crystallized ligand of 7S3F) | ODC1 | -4.23 ± 0.12 |
| 1 | Gamma-Mangostin | IDHC | -6.93 ± 0.3 |
| 2 | (7R)-1-(4-fluorobenzyl)-N-{3-[(1S)-1-hydroxyethyl]phenyl}-7-methyl-5-(1H-pyrrol-2-ylcarbonyl)-4,5,6,7-tetrahydro-1H-pyrazolo[4,3-c]pyridine-3-carboxamide (59D: Co-crystallized ligand of 5DE1) | IDHC | -8.91 ± 0.27 |
| 1 | Ent-Epicatechin | NOS3 | -5.23 ± 0.18 |
| 2 | 7-{3-(aminomethyl)-4-[(pyridin-3-yl)methoxy]phenyl}-4-methylquinolin-2-amine (OUS: Co-crystallized ligand of 6PP4) | NOS3 | -6.55 ± 0.08 |
| 1 | Bolinaquinone | PA2GA | -6.05 ± 0.41 |
| 2 | 3-(5'-Benzyl-2'-Carbamoylbiphenyl-3-Yl)Propanoic Acid (X28: Co-crystallized ligand of 5G3N) | PA2GA | -7.33 ± 0.4 |
| 1 | (1R,2S,7S,8S,9S,10S)-2,6,6,9-Tetramethyltetracyclo[5.4.0.0²,⁹.0⁸,¹⁰]Undecane | UD2B7 | -5.4 ± 0.07 |
| 2 | (1R,2R,7S,9S)-3,3,7-Trimethyl-8-Methylidenetricyclo[5.4.0.0²,⁹]Undecane | UD2B7 | -5.44 ± 0.14 |
| 3 | (1aR,4S,4aR,7S,7aS,7bS)-1,1,4,7-tetramethyl-2,3,4a,5,6,7,7a,7b-octahydro-1aH-cyclopropa[e]azulen-4-ol | UD2B7 | -5.93 ± 0.23 |
| 4 | (1R,2S,7S,8S,9R)-2,6,6,9-Tetramethyltricyclo[5.4.0.0²,⁹]Undecan-8-Ol | UD2B7 | -5.63 ± 0.21 |
| 5 | (1R,2S,7S,8R,9R)-2,6,6,9-Tetramethyltricyclo[5.4.0.0²,⁹]Undecan-8-Ol | UD2B7 | -5.7 ± 0.21 |
| 6 | (1S,2R,5S,6S,7S,8R)-1,5-Dimethyl-8-(Prop-1-En-2-Yl)Tricyclo[5.3.0.0²,⁶]Decane | UD2B7 | -5.78 ± 0.19 |
| 7 | (1S,2R,5S,6S,7S,8S)-1,5-Dimethyl-8-(Prop-1-En-2-Yl)Tricyclo[5.3.0.0²,⁶]Decane | UD2B7 | -5.85 ± 0.17 |
| 8 | (+)-Ledol | UD2B7 | -5.77 ± 0.18 |
| 9 | (1S,2R,7S,8R,9S)-2,6,6,9-tetramethyltricyclo[5.4.0.02,8]undecan-9-ol | UD2B7 | -5.9 ± 0.24 |
| 10 | (1S,2S,7S,8S)-2,6,6,9-Tetramethyltricyclo[5.4.0.0²,⁸]Undec-9-Ene | UD2B7 | -5.52 ± 0.1 |
| 11 | Epiglobulol | UD2B7 | -5.78 ± 0.17 |
| 1 | 1-benzyl-1H-imidazole | HMOX1 | -4.69 ± 0.09 |
| 2 | Climbazole | HMOX1 | -6.2 ± 0.1 |
| 3 | 2-(1H-imidazol-1-yl)acetic acid | HMOX1 | -4.5 ± 0.11 |
| 4 | 3-{4-[(1H-imidazol-1-yl)methyl]phenyl}prop-2-enoic acid | HMOX1 | -5.5 ± 0.12 |
| 5 | 3-(1H-imidazol-1-yl)-2-oxopropanoic acid | HMOX1 | -4.76 ± 0.13 |
| 6 | Imidazolepropionic Acid | HMOX1 | -4.71 ± 0.12 |
| 7 | [1-hydroxy-2-(1H-imidazol-1-yl)-1-phosphonoethyl]phosphonic acid | HMOX1 | -5.4 ± 0.22 |
| 8 | 1-(3-imidazol-1-ylpropyl)-5-(2-methylpropyl)-4-phenyl-imidazole (B5B: Co-crystallized ligand of 6EHA) | HMOX1 | -6.91 ± 0.22 |
| 1 | 2-benzyl-8-ethoxy-1,3-dimethyl-2H,4H-cyclohepta[c]pyrrol-4-one | PA2G5 | -6.74 ± 0.19 |
| 1 | 2-methanesulfonyl-6-{1-methanesulfonyl-5H,6H,7H,8H,9H-cyclohepta[c]pyridin-3-yl}pyridine | SDHB | -5.33 ± 0.21 |
| 1 | Isosorbide mononitrate | GSR | -5.37 ± 0.19 |
